# The *Arabidopsis* COX11 Homolog is Essential for Cytochrome *c* Oxidase Activity

**DOI:** 10.3389/fpls.2015.01091

**Published:** 2015-12-18

**Authors:** Ivan Radin, Natanael Mansilla, Gerhard Rödel, Iris Steinebrunner

**Affiliations:** ^1^Institute for Genetics, Department of Biology, Technische Universität DresdenDresden, Germany; ^2^Instituto de Agrobiotecnología del Litoral-Consejo Nacional de Investigaciones Científicas y Técnicas, Universidad Nacional del LitoralSanta Fe, Argentina; ^3^Department of Biology, Technische Universität DresdenDresden, Germany

**Keywords:** COX11 (cytochrome *c* oxidase 11), copper chaperone, mitochondria, COX complex, root development, pollen development, *Arabidopsis thaliana*

## Abstract

Members of the ubiquitous COX11 (cytochrome *c* oxidase 11) protein family are involved in copper delivery to the COX complex. In this work, we characterize the *Arabidopsis thaliana* COX11 homolog (encoded by locus At1g02410). Western blot analyses and confocal microscopy identified *Arabidopsis* COX11 as an integral mitochondrial protein. Despite sharing high sequence and structural similarities, the *Arabidopsis* COX11 is not able to functionally replace the *Saccharomyces cerevisiae* COX11 homolog. Nevertheless, further analysis confirmed the hypothesis that *Arabidopsis* COX11 is essential for COX activity. Disturbance of *COX11* expression through knockdown (KD) or overexpression (OE) affected COX activity. In KD lines, the activity was reduced by ~50%, resulting in root growth inhibition, smaller rosettes and leaf curling. In OE lines, the reduction was less pronounced (~80% of the wild type), still resulting in root growth inhibition. Additionally, pollen germination was impaired in *COX11* KD and OE plants. This effect on pollen germination can only partially be attributed to COX deficiency and may indicate a possible auxiliary role of COX11 in ROS metabolism. In agreement with its role in energy production, the *COX11* promoter is highly active in cells and tissues with high-energy demand for example shoot and root meristems, or vascular tissues of source and sink organs. In *COX11* KD lines, the expression of the plasma-membrane copper transporter *COPT2* and of several copper chaperones was altered, indicative of a retrograde signaling pathway pertinent to copper homeostasis. Based on our data, we postulate that COX11 is a mitochondrial chaperone, which plays an important role for plant growth and pollen germination as an essential COX complex assembly factor.

## Introduction

The cytochrome *c* oxidase (COX) is a crucial component of the mitochondrial respiratory chain, which is of utmost importance for providing cellular energy. Dysfunction of COX inevitably brings processes that depend on respiration-based energy supply to a sudden halt: respective *Saccharomyces (S.) cerevisiae* mutants stop growing on non-fermentable carbon sources (Merz and Westermann, 2009) as do COX-deficient *Chlamydomonas reinhardtii* mutants under heterotrophic conditions (Colin et al., 1995). In *Arabidopsis thaliana*, compromised COX function prevents development beyond the embryo-stage (Attallah et al., 2011; Steinebrunner et al., 2011; Welchen et al., 2012; Dahan et al., 2014).

The multi-subunit COX harbors two copper (Cu) centers, which are essential for the electron transfer through the complex to molecular oxygen (reviewed in Khalimonchuk and Rödel, 2005). One of the copper centers, Cu_A_, lies in the subunit COX2, while the other one, Cu_B_, is formed in the subunit COX1. The Cu ions for Cu_A_ and Cu_B_ are provided by the two copper chaperones SCO1 (for synthesis of cytochrome *c* oxidase 1) (Schulze and Rödel, 1988; Lode et al., 2000; Nittis et al., 2001) and COX11 (Banting and Glerum, 2006), respectively. Analysis of *Rhodobacter (R.) sphaeroides* mutants, which lack the *COX11* gene, confirms that the encoded protein is required for the insertion of Cu into the Cu_B_ site (Hiser et al., 2000). Yeast COX11 is N-terminally anchored in the inner mitochondrial membrane with the majority of the protein protruding into the intermembrane space (IMS) (Carr et al., 2005; Khalimonchuk et al., 2005). *In vitro* studies with SCO1 and COX11 from *S. cerevisiae* indicate that the two proteins most likely receive the Cu ions from the small soluble copper chaperone COX17 located in the IMS (Horng et al., 2004). While evidence was presented that the Cu transfer from COX11 to COX1 may occur co-translationally (Khalimonchuk et al., 2005), the exact molecular mechanism of the transfer is unknown. However, the Cu-binding mode has been well studied: COX11 forms a homodimer that binds two Cu ions, each of which is ligated by three cysteine residues (Carr et al., 2002). Based on the structure of the soluble *Sinorhizobium meliloti* COX11, it was suggested that two of these cysteines are derived from the conserved Cu-binding motif CxC of one subunit, while the third cysteine is provided by the respective Cu-binding motif of the second subunit (Banci et al., 2004). The exact role of a third conserved cysteine outside of the Cu-binding motif that is essential for COX11 function (Banting and Glerum, 2006) is less clear. Data obtained with COX11 from *R. sphaeroides* suggest that it might serve as a transient Cu-binding site during Cu insertion into COX1 (Thompson et al., 2010). *S. cerevisiae cox11* null mutants are respiratory deficient (Tzagoloff et al., 1990; Carr et al., 2002; Banting and Glerum, 2006), in agreement with the essential contribution of COX11 for COX assembly.

*COX11* is a ubiquitous gene found in several bacterial genomes and every eukaryotic genome publicly available. However, no data on COX11 function exist for plants. Here, we close this gap by presenting an extensive characterization of the COX11 homolog in *Arabidopsis*, which includes expression pattern, subcellular localization, knockdown as well as overexpression effects. We show that COX11 is essential for COX activity and that it affects vital physiological processes such as pollen germination, development, and growth.

## Materials and methods

### Plant material and culture conditions

*A. thaliana* Columbia (Col) 0 was used as the WT and background for all mutants generated. Plants with the silenced *COX11* gene were generated using an amiRNA designed with the online tool WMD3 (Web MicroRNA Designer; wmd3.weigelworld.org) (Ossowski et al., 2008). Overlap extension PCR (Pogulis et al., 1996) with specific primers (Supplementary Table 1) was used to create the amiRNA precursor for the selected target as described in WMD3 (Supplementary Figure 1). The vector pNB47 (Bologna et al., 2009) was kindly provided by Javier Palatnik (Institute of Molecular and Cellular Biology, Rosario, Argentina). This binary vector provides the precursor sequence for the miR319a as the template for overlap extension PCR and allows amiRNA expression in the plant under the CaMV *35S* promoter. The PCR product was cloned into the pNB47 using the *Pst*I and *Bam*HI restriction sites, which replaced the miR319a precursor sequence. *Arabidopsis* plants were transformed by the floral dip procedure (Clough and Bent, 1998). Selected plants were confirmed by PCR using specific primers (Supplementary Table 1).

Two independent T2-generation lines, KD1 and KD2, were obtained. The KD2 line was used in the T2 generation only, because the KD effect was lost in the T3 generation (Supplementary Figure 2A). From the KD1 line, two separate seed batches (KD1-1 and KD1-2; progeny of two individual T2 generation plants) were harvested and used in the T3 generation unless stated otherwise. The T-DNA insertion lines for the locus At1g02410 [Supplementary Figure 3; SALK_105793, SALK_003445C (Alonso et al., 2003), SAIL_603_G12, SAIL_861_D09, SAIL_683_B03 (McElver et al., 2001)] were provided by The Nottingham *Arabidopsis* Stock Centre and *Arabidopsis* Biological Resource Center. Presence and positions of the T-DNA insertion were confirmed by PCR with T-DNA and genomic DNA specific primers (listed in Supplementary Table 1).

All other constructs were cloned applying the Gateway cloning technology (LifeTechnologies, USA) according to the manufacturer's instructions. Primers used for cloning are listed in the Supplementary Table 1. The plasmid pENRT223-*COX11* (Yamada et al., 2003), or clone G61127, obtained from *Arabidopsis* Biological Resource Center, was used as a template or starting plasmid for all constructs. For localizations experiments, the *COX11* cDNA was inserted into the pGWB553 destination vector (Nakagawa et al., 2007) to generate the *35S*:*COX11*-*mRFP* (monomeric red fluorescent protein) construct. To generate the *35S*:*mRFP* construct, the *mRFP* cDNA was inserted into the pGWB502 destination vector (Nakagawa et al., 2007). For generation of the GUS lines, the putative *COX11* promoter region (−419 to +366 bp with the transcription start site being +1) was recombined into the pMDC163 plasmid (Curtis and Grossniklaus, 2003). For *COX11* overexpression lines (OE), the *COX11* cDNA was cloned into the pGWB514 and pGWB502 (Nakagawa et al., 2007) destination vectors to obtain the *35S*:*COX11-3HA* (OE1) and *35S*:*COX11* (OE2) constructs, respectively. For both OE lines, T3-generation homozygous lines were selected and used for all experiments, except for the experiments shown in **Figure 3**, for which hemizygous T3-generation *35S*:COX11*-3HA* plants were used. Constructs were generally transformed into Col-0 background via the floral dip method (Clough and Bent, 1998). Only the two mRFP lines *35S*:*COX11*-*mRFP* and *35S*:*mRFP* were also transformed into the plant background expressing green fluorescent protein targeted to mitochondria (mt-GFP) (Nelson et al., 2007). The genomic T-DNA insertion for all constructs, except for *COX11:GUS*, were confirmed by PCR with primers specific for individual constructs (listed in Supplementary Table 1). The *COX11:GUS* insertion was confirmed by direct GUS staining of seedlings.

Plants were grown on sterile selective or non-selective Murashige and Skoog (MS) + sucrose (1 or 2%) plates. For selection, 30 or 50 μg mL^−1^ of kanamycin and/or 20 μg mL^−1^ of hygromycin were added. Alternatively, plants were cultured on soil [Einheitserde, type P, Pätzer, Sinntal-Jossa, Germany; mixed with sand 4:1, fertilized by watering with 0.1% (v/v) Wuxal Basis, Aglukon].

Plants were cultured in growth chamber with light intensity of 150 μmol m^−2^ s^−1^, relative humidity of 35% and day/night temperatures of 24/21°C, respectively. Two types of day/night cycles were used: long day (16-h day) and short day (10-h day).

### Yeast material and culture conditions

*S. cerevisiae* wild-type (WT) strain BY4741 [Accession no. Y00000; *MATa, his3*Δ*1, leu2*Δ*0, met15*Δ*0, ura3*Δ*0, (rho*^+^*)] and* Δ*cox11* deletion strain [Accession no. Y06479; *MATa, his3*Δ*1, leu2*Δ*0, met15*Δ*0, ura3*Δ*0, cox11::kanMX4, (rho*^+^*)*] were obtained from EUROSCARF (Frankfurt, Germany). For complementation assays, the full-length yeast and *Arabidopsis COX11* sequences were inserted by Gateway cloning into yeast expression vectors (Alberti et al., 2007) [pAG415ADH-ccdB-3HA (for moderate overexpression, single copy vector with *ADH* promoter), pAG425ADH-ccdB-3HA (for strong overexpression, high copy vector with *ADH* promoter) and pAG415COX11-ccdB-3HA (for native expression, single copy vector in which the *ADH* promoter was replaced by 1500 bp of the *COX11* promoter region)]. Two chimeric proteins (CHYM-1 and -2) were generated by overlap extension PCR (Pogulis et al., 1996) and inserted in the same expression vectors. Cloning primers are listed in Supplementary Table 1. Yeast cells were transformed as described in Gietz and Schiestl (2007).

Transformed yeast strains were cultured on minimal media [0.5% (w/v) ammonium sulfate, 0.19% (w/v) yeast nitrogen bases, 2% (w/v) glucose, 2.5% (w/v) agar, and selection amino acids]. For complementation assays, strains were spotted on either YPD [1% (w/v) yeast extract, 2% (w/v) peptone, 2% (w/v) glucose, 2% (w/v) agar] or YPEG [1% (w/v) yeast extract, 2% (w/v) peptone, 2% (w/v) glycerol, 3% (v/v) ethanol, 2% (w/v) agar] media. Copper was added to the YPEG media at the concentration 0.5 mM, which according to the literature is sub-toxic (Liang and Zhou, 2007). For spotting, yeast strains were inoculated into liquid minimal media and upon reaching the stationary phase, the cultures were sequentially diluted from 1.25 × 10^6^ to 1.25 × 10^3^ cells/ml in four steps (The assumption was that the OD_600_ of 0.1 equals 1 × 10^6^ cells/ml). From these dilution series, 8 μl of each dilution were spotted on the appropriate solid media plate. Plates were incubated at 30°C for 2 days (YPD media), 4 days [WT and Δ+*Sc COX11* (= Δ*cox*11 strain transformed with the expression vector containing the yeast *COX11* gene) strains on YPEG] and 20 days (other strains on YPEG).

### Bioinformatic analysis

For single and multiple protein sequence alignments, the EMBOSS Needle and Cluster Omega software (The European Bioinformatics Institute) (McWilliam et al., 2013), respectively, were used. For the prediction of cellular targeting and targeting signal cleavage sites, the programs TargetP (Emanuelsson et al., 2000) and MitoProt II (Claros and Vincens, 1996) were used. Transmembrane domains were predicted with the TMHMM2.0 (Krogh et al., 2001). The prediction of the proteins' secondary structures was performed with PSIPRED (McGuffin et al., 2000). *Arabidopsis* sequence information was obtained from The *Arabidopsis* Information Resource (Lamesch et al., 2012), while all other sequences were retrieved from the GeneBank (Benson et al., 2013).

### Confocal microscopy

Confocal imaging was performed on a Zeiss LSM780 upright microscope of the Light Microscopy Facility, a core facility of the BIOTEC/CRTD at Technische Universität Dresden. Colocalization images were made with the C-Apochromat 63 × /1.20 W korr M27 objective. GFP and mRFP were simultaneously excited with 488 and 561-nm lasers, respectively and their fluorescences simultaneously detected in the range of 470 and 552 nm for GFP, and 575 and 650 nm for mRFP. Image analysis was performed with the Zeiss Zen2012 software (also used for acquisition) and the colocalization plugin JACoP (Bolte and Cordelières, 2006) of the Fiji software (Schindelin et al., 2012). For colocalization studies, 14-day-old seedlings (cultured on selective MS + 2% sucrose plates under long-day conditions) were imaged with water as imaging medium.

### Protein and mitochondria isolation

For total protein isolation, *Arabidopsis* leaves from plants cultured on soil under short-day condition were mixed with 2 mL of QB buffer [100 mM potassium phosphate buffer pH 7.8, 1 mM EDTA, 1% (v/v) Triton X-100, 10% (v/v) glycerol, 1 mM DTT] (Ni et al., 1996) per 1 g of tissue and ground with a mortar and pestle. Samples were centrifuged at 14,000 g for 15 min at 4°C. The supernatant was saved and stored at −80°C.

Crude yeast mitochondria preparations used in the Supplementary Figure 6 were isolated as previously described (Gey et al., 2014). For mitochondria isolation, yeast strains were cultured in minimal media.

Crude *Arabidopsis* mitochondria preparations used in the Supplementary Figure 9 were isolated from etiolated seedlings as previously described (Steinebrunner et al., 2011). Crude mitochondria for the COX activity assay and the BN-PAGE (**Figure 6**), and pure mitochondria for the sodium carbonate extraction procedure (**Figure 3**) were isolated from leaves of plants cultured in short day as described in Keech et al. (2005). For the isolation of pure mitochondria, one modification was made: Cysteine was replaced in the grinding buffer with 0.005% (v/v) β-mercaptoethanol. Protein concentrations were determined with the DC protein assay kit from Bio-Rad (USA).

### SDS-PAGE and Western blot analysis

For protein separation, the gradient (4–12%) NuPAGE electrophoresis system with the NuPAGE MOPS buffer from LifeTechnologies (USA) was used. After separation, proteins were transferred onto a polyvinylidene difluoride (PVDF) membrane (Immobilion-P, Millipore, USA) with a semi-dry blotter from Peqlab (Germany). For Western blot detection, ECL prime Western blotting detection reagents from GE Healthcare (UK) were used. Most primary antibodies were purchased from Agrisera, Sweden [cytosolic fructose-1,6-bisphosphatase (cFBP, 1:5000); cytochrome *c* oxidase subunit 2 (COX2, 1:2000); glycine decarboxylase complex subunit H (GDCH, 1:5000); rubisco large subunit (RbCL, 1:10,000); voltage-dependent anion-selective channel 1 (VDAC1, 1:10,000)]. Anti-HA and anti-GFP (both 1:1000) were obtained from Roche (Switzerland), while anti-mRFP (1:5000) was purchased from Rockland (USA). Antibodies against yeast phosphoglycerate kinase 1 (PGK1, 1:1000) and porin 1 (POR1, 1:1000) were obtained from LifeTechnologies (USA). Dilutions of the antibodies are indicated in parentheses. Anti-mouse and anti-rabbit horseradish peroxidase (HRP)-coupled secondary antibodies (1:10,000) were obtained from GE healthcare (UK). Between detections, the membranes were stripped by one 30-min incubation in stripping buffer [62.5 mM Tris-Cl pH 6.7, 2% SDS (w/v) and freshly added 100 mM β-mercaptoethanol] at 55°C.

### Sodium carbonate extraction

Sodium carbonate extraction was performed as previously described (Fujiki et al., 1982), with some modifications. Pure mitochondria (100 μg each) were spun down with 15,000 g for 15 min at 4°C, resuspended in 500 μL of 0.2 M Na_2_CO_3_ and kept on ice for 1 h. The suspension was centrifuged, 220,000 g (max) for 1 h at 4°C. The pellet was resuspended in 10 mM Tris-Cl pH 7.6, while the supernatant was precipitated with TCA [trichloroacetic acid, final concentration of 10% (v/v)] over night at −25°C. Proteins were pelleted by centrifugation at 20,000 g for 1 h at 4°C. The pellet was rinsed with ice-cold 80% (v/v) acetone, spun down, dried, and finally resuspended in 10 mM Tris-Cl pH 7.6.

### COX complex activity measurement

COX complex activity was determined by monitoring the oxidation of reduced cytochrome *c* by crude mitochondria as previously described (Wigge and Gardeström, 1987). Because the crude mitochondria fractions had varying degrees of purity, the citrate synthase activity was also determined for each fraction and used for normalization of COX activity as introduced in Steinebrunner et al. (2014).

### Blue-native PAGE and in-gel COX activity staining

Protein complexes present in crude mitochondria fractions were separated on a gradient blue-native (BN)-PAGE gel (3–13%, with a 3% stacking gel) as previously described (Schägger and Jagow, 1991; Steinebrunner et al., 2014). The in-gel COX activity staining was performed as described in Steinebrunner et al. (2014).

### RNA isolation and quantitative real-time RT-PCR (qPCR)

Total RNA was isolated from 50 to 75 mg of 14-day-old seedlings (cultured under long-day conditions on MS + 1% sucrose plates) with the RNeasy plant mini kit (Qiagen, Germany). Genomic DNA contaminations were removed with the on-column RNase-free DNase set (Qiagen, Germany). RNA quantity and quality were assessed using the Nanodrop ND-1000 (Peqlab, Germany) and BioAnalyser 2100 (Agilent, USA) device, respectively. The RNA integrity numbers (RIN) for most samples were in the range of 7.5–8.5. Total RNA (500 ng) was reverse transcribed with oligo(dT)_18_ primers and the RevertAid first strand cDNA synthesis kit (Thermo Fisher Scientific, USA). Of all samples, control reverse transcription reactions without enzyme were set up to check for genomic DNA contaminations.

qPCR was performed with the 2 × DyNAmo ColorFlash SYBR Green qPCR Master Mix from Thermo Fisher Scientific (USA). A 50-fold dilution of cDNA was used as a template. The efficiency and optimal concentrations of all primer pairs were experimentally determined (Supplementary Table 2). The following cycling conditions were selected for all reactions: 1 × [95°C/8 min], 40 × [95°C/10 s; 60°C/20 s; 72°C/15 s, plate read], 1 × [95°C/10 s], melting curve from 65 to 95°C with a heating ramp of 0.5°C/5 s. The qPCR reactions were always run in triplicates in a C1000 thermo-cycler and CFX96 real-time system (Bio-Rad, USA). White non-skirted 96-well “low profile” PCR plates (Thermo Fisher Scientific, USA) were used and sealed with absolute qPCR film (Thermo Fisher Scientific, USA). The data was statistically analyzed with the Bio-Rad CFX Manager 3.1 software. Transcript levels were normalized with both *actin 2* (*ACT2*) and *protein phosphatase 2A subunit A3* (*PP2AA3*).

### Root growth assay

For root length measurements, seedlings were cultured on MS plates + 1% (w/v) sucrose without antibiotics. In the heterozygous KD lines, WT seedlings, which appeared in the expected Mendelian ratios, could be easily distinguished from KD seedlings by their twice as long roots and were removed from the analysis. All seedlings were cultured in parallel. The mutant lines, however, were divided into three groups (KD1-1 and KD1-2/KD2/OE1 and OE2) and cultured on separate plates, but always with the WT on one-half of the same plate. The root lengths were normalized to WT from the same plate. For treatments, the chemicals were added to the medium in the desired concentration before plate pouring. Plates were kept in the vertical position under long-day conditions for 12 days. Each plate was photographed with the Olympus digital camera C-5000 Zoom, and root lengths were determined with the Root Detection 0.1.2 software (http://labutils.de/).

### Pollen viability assay

Flowers were harvested from 10-week-old *Arabidopsis* plants grown under long-day conditions. Seeds were air-dried for 2 h and then slowly rehydrated in a humid chamber for 45 min at 30°C. Flowers were gently dipped into working solution [17% (w/v) sucrose with 2 μg mL^−1^ of FDA (fluorescein diacetate) (Heslop-Harrison and Heslop-Harrison, 1970)] to release the pollen. After 15 min, the pollen grains were observed in the same media under a fluorescence microscope (Axio Observer Z1 inverted microscope with AxioCamMR3, from Zeiss) with EC Plan-Neofluar 10 × /0.30 Ph1 objective (Zeiss) and 38 HE Green Fluorescence Reflector (Zeiss). For image acquisition and analysis, AxioVision Rev 4.8 software was used. Fluorescent pollen grains were counted as viable.

### Pollen germination assay

Flowers were prepared as described for the pollen viability assay above. Pollen was germinated in duplicates or triplicates with the sitting-drop method (Bou Daher et al., 2009). In the appropriate experiments, KCN was added directly to the germination media.

### GUS staining

GUS activity staining was performed as previously described (Wolf et al., 2007). For the assay, *COX11*:*GUS* plants were cultured either on MS + 2% sucrose plates or on soil under long-day conditions. For determination of GUS activity in pollen, x-gluc (5-bromo-4-chloro-3-indolyl-b-D-glucuronide) was added to a final concentration of 1 mM either directly to germination media (Bou Daher et al., 2009) or to 18% (w/v) sucrose for pollen rehydration. Pollen was germinated or rehydrated with the sitting-drop method for 4 h at 30°C and subsequently transferred to 37°C for over-night incubation.

### Copper content measurement

The copper content was determined in leaves of 9-week-old plants cultured under short-day conditions by Inductively Coupled Plasma Optical Emission Spectrometry (ICP-OES). For ICP-OES analysis, samples were prepared as previously described (Cohu and Pilon, 2007; Tan et al., 2010).

## Results

### Identification of a COX11 family member in *Arabidopsis*

**Figure 1:**
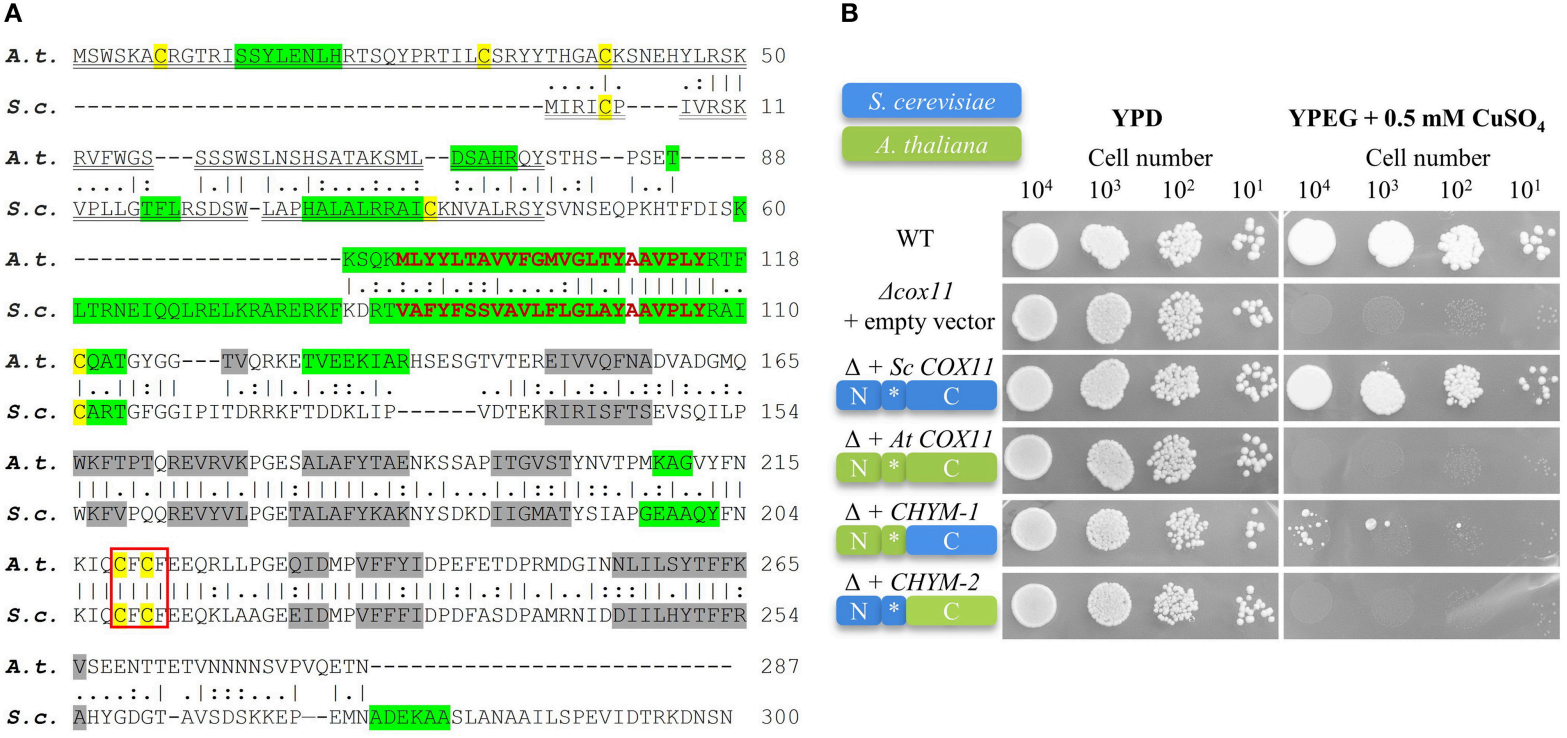
**The ***Arabidopsis*** COX11 homolog shares structural and sequence similarities with yeast COX11, but cannot functionally complement it. (A)** The *A. thaliana* (A. t.) and *S. cerevisiae* (S. c.) protein sequence were retrieved from the GenBank database and aligned. The vertical lines (I) indicate identical residues between the two sequences, while the colons (:) and periods (.) represent conservative substitutions with strongly and weakly similar properties, respectively. Cysteines are labeled yellow. The putative copper-binding motifs are boxed. The predicted cleavable N-terminal targeting signals are double underlined. The predicted transmembrane domains are shown in bold red letters, while the predicted α-helices and β-sheets are highlighted in green and gray, respectively. **(B)** Results from a respiratory competence test of yeast Δ*cox11* stains moderately overexpressing either full-length *S. cerevisiae COX11, Arabidopsis COX11*, or sequences encoding the chimeric proteins CHYM-1 or -2. Schematic diagrams of the proteins depict their composition (N, N-terminal region; C, C-terminal region; ^*^, transmembrane domain) and the origin of the respective regions (*S. cerevisiae*, blue; *A. thaliana*, green). Serial dilutions of yeast strains were spotted on fermentable glucose media (YPD) or respiratory media (YPEG, ethanol + glycerol media supplemented with 0.5 mM CuSO_4_).

To identify members of the COX11 family, the *Arabidopsis* Information Research (TAIR) genome database was searched for genes with sequence similarity to *COX11* genes from other species. A single candidate locus (At1g02410) was identified. The At1g02410 locus is transcribed and predicted to code for a 287-amino acid protein. The amino acid sequence of the *Arabidopsis* COX11 homolog (further referred to as *Arabidopsis* COX11) shares 32% identity and 45% similarity to the COX11 protein of *S. cerevisiae* (Carr et al., 2005; Banting and Glerum, 2006) (Figure 1A).

The yeast COX11 protein was shown to carry an N-terminal mitochondrial targeting sequence. In agreement with this, the *Arabidopsis* COX11 homolog is predicted to be targeted to mitochondria (0.711 and 0.93 probability according to the programs TargetP and MitoProtII, respectively), and to possess a cleavable N-terminal mitochondrial targeting signal (Figure 1A). Both, the yeast and the *Arabidopsis* COX11 protein are predicted to harbor a single transmembrane domain of 23 amino acids close to their N-terminal ends (Figure 1A). Interestingly, predicted secondary structures of the two proteins, particularly regarding the numbers and positions of β-sheets in C-terminal parts, also show high similarity (Figure 1A). The *Arabidopsis* COX11 protein contains six cysteines, with the N-terminal three being part of the putative cleavable mitochondrial targeting signal and hence probably not present in the mature protein. However, the remaining three cysteines are conserved among COX11 family members (Supplementary Figure 4) and have been shown to be essential for the function of yeast COX11 (Banting and Glerum, 2006). Cysteines C_219_ and C_221_ (*Arabidopsis* numbering) are part of the conserved copper-binding motif CFCF.

### *Arabidopsis COX11* cannot functionally complement a *COX11* deletion strain of *Saccharomyces cerevisiae*

We tested whether the *Arabidopsis* homolog can functionally replace its *S. cerevisiae* counterpart and rescue the respiratory-deficient phenotype of yeast Δ*cox11* strain (Figure 1B). We cloned the full-length yeast and *Arabidopsis COX11* coding sequences, as well as the sequences encoding two chimeric proteins, generated from parts of both homologs into yeast expression vectors. In CHYM-1, the C-terminal region was derived from yeast COX11 (containing the Cu-binding motif), while the transmembrane anchor and the N-terminal region including the mitochondrial targeting sequence stemmed from *Arabidopsis* COX11. In the other chimera (CHYM-2), the origin of the sequences was vice versa. The exchange site was between V_112_ and P_113_ (*Arabidopsis* numbering, Figure 1A).

Yeast Δ*cox11* strains that moderately overexpressed the respective constructs, were tested for respiratory competence (Figure 1B). The control (full-length yeast *COX11*) was able to restore respiratory competence to WT levels, while all other constructs failed to do so. The only exception was noted upon transformation with the *CHYM-1* construct that resulted in a weak and sporadic growth after long incubation (more than 20 days). Parallel spotting on fermentable glucose media (YPD) demonstrated viability of all yeast strains. The same results were obtained with native expression or strong overexpression of the various constructs (Supplementary Figure 5). All four constructs, irrespective of the origin of their targeting signal (yeast or *Arabidopsis*), were almost completely targeted to mitochondria (Supplementary Figure 6). Hence, incorrect intracellular targeting of the proteins can be excluded as the reason for the observed failure to complement the Δ*cox11* strain. In summary, despite their high similarity, the *Arabidopsis* COX11 protein cannot functionally replace its yeast counterpart.

### *Arabidopsis* COX11 is localized to mitochondria

**Figure 2:**
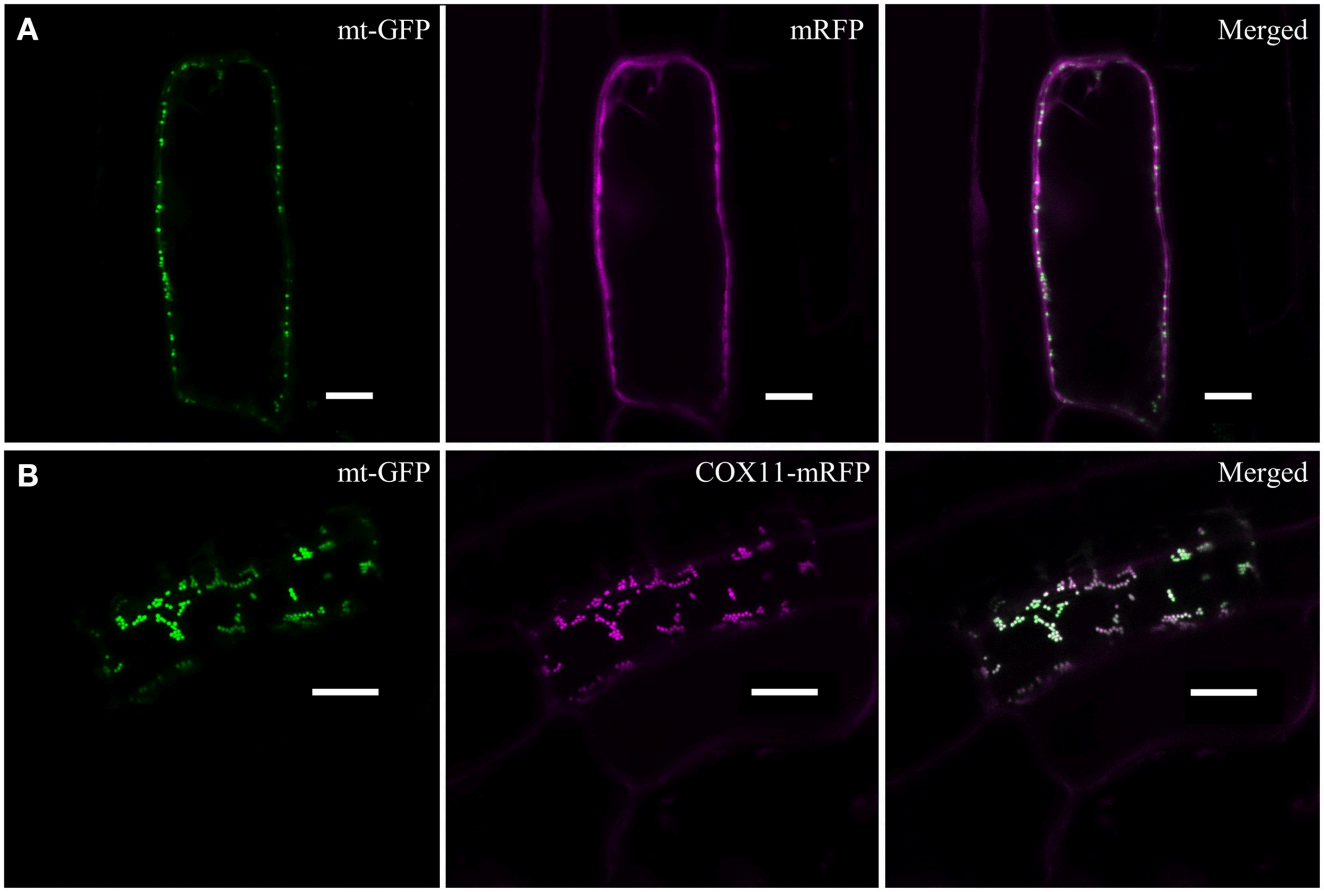
**COX11-mRFP is localized to mitochondria**. LSM images of root cells from *Arabidopsis* lines with GFP-labeled mitochondria, expressing either *mRFP* alone **(A)** or *COX11-mRFP* **(B)**, are shown. The fluorescence of mRFP and GFP targeted to mitochondria (mt-GFP) was falsely colored magenta and green, respectively. Scale bars correspond to 10 μm each.

In order to examine the cellular localization of *Arabidopsis* COX11, its ORF was fused at its 3′ end to the sequence encoding mRFP and transformed into an *Arabidopsis* line expressing mitochondria-targeted GFP (mt-GFP) (Nelson et al., 2007). As a control, mRFP alone was expressed in the same genetic background. As expected, mRFP was detected in the cytoplasm of the control lines (Figure 2A). Upon fusion to COX11, however, mRFP co-localized with mt-GFP-labeled mitochondria (Figure 2B), demonstrating that COX11 contains sequence information for mitochondrial targeting.

Imaging plants expressing only mt-GFP or mRFP with the same settings as used for the colocalization images ruled out a possible bleed-through between the GFP and mRFP channels (Supplementary Figure 7). No overlap between GFP and mRFP signals was detected. In addition, colocalization of COX11-mRFP and mt-GFP was evaluated by scatter-plotting of the two channel images (Supplementary Figure 8) and calculation of the colocalization coefficients (Supplementary Table 3). Western blot analyses of cell fractions (Supplementary Figure 9) corroborated the mitochondrial localization of COX11-mRFP.

**Figure 3:**
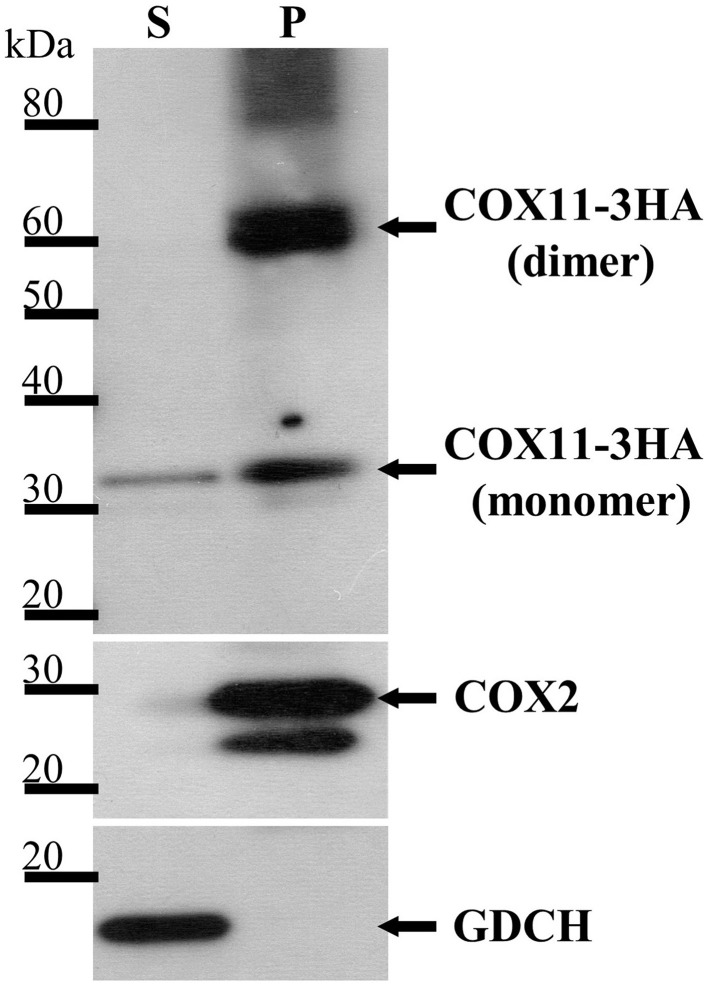
**COX11-3HA is an integral membrane protein**. Pure mitochondria were isolated from plants expressing *COX11-3HA* and subjected to Na_2_CO_3_ treatment to separate soluble (S) from membrane and membrane-associated proteins, which remained in the pellet (P). The S and P fractions (from 100 μg of mitochondria) were analyzed by Western blotting, using antibodies against HA (from human influenza hemagglutinin) to detect COX11-3HA of the theoretical molecular weight of 30 kDa after cleavage of the predicted targeting signal. The purity of the fractions was assessed by detection of the marker COX2 (cytochrome *c* oxidase subunit 2, theoretical molecular weight of 30 kDa) for integral and GDCH (glycine decarboxylation complex subunit H, theoretical molecular weight of 16 kDa) for soluble proteins. All proteins were detected on the same membrane, which was stripped between each detection.

In order to determine, whether *Arabidopsis* COX11 is a soluble or an integral membrane protein, pure mitochondria from a WT line expressing 3HA-tagged *COX11* were treated with sodium carbonate. Upon centrifugation, the two fractions containing the integral membrane and the soluble proteins were subjected to a Western blot analysis with HA-antibody. Figure 3 shows that a protein of approximately 30 kDa corresponding to the mature COX11-3HA protein was mainly present in the insoluble pellet fraction, in agreement with the prediction of an integral membrane protein. Another prominent protein in the pellet fraction of about 60 kDa suggests COX11 dimer formation, as was shown for yeast COX11 (Carr et al., 2002). In addition, high molecular-weight aggregates were observed. The persistence of the COX11 dimers on SDS-PAGE gels is quite intriguing (Figure 3 and Supplementary Figures 6, 9). Possibly the dimer stability results from the strong binding of binuclear Cu(I) cluster, which bridges the COX11 monomers (see Introduction). As outlined in a recent review (Palm-Espling et al., 2012), copper clusters often exhibit an extraordinary stability against chemical and thermal unfolding.

The small amount of COX11-3HA that was detected in the soluble fraction cannot be accounted for by cross-contamination of the two fractions, as the membrane marker COX2 (cytochrome *c* oxidase subunit 2) and the soluble marker GDCH (glycine decarboxylase complex subunit H) were exclusively present in the pellet and supernatant fractions, respectively. It remains unclear whether the soluble portion of COX11 reflects the physiological situation, or results from the artificial high-level expression of *COX11-3HA*.

### The *COX11* promoter is active in tissues with high metabolic and/or division rates

**Figure 4:**
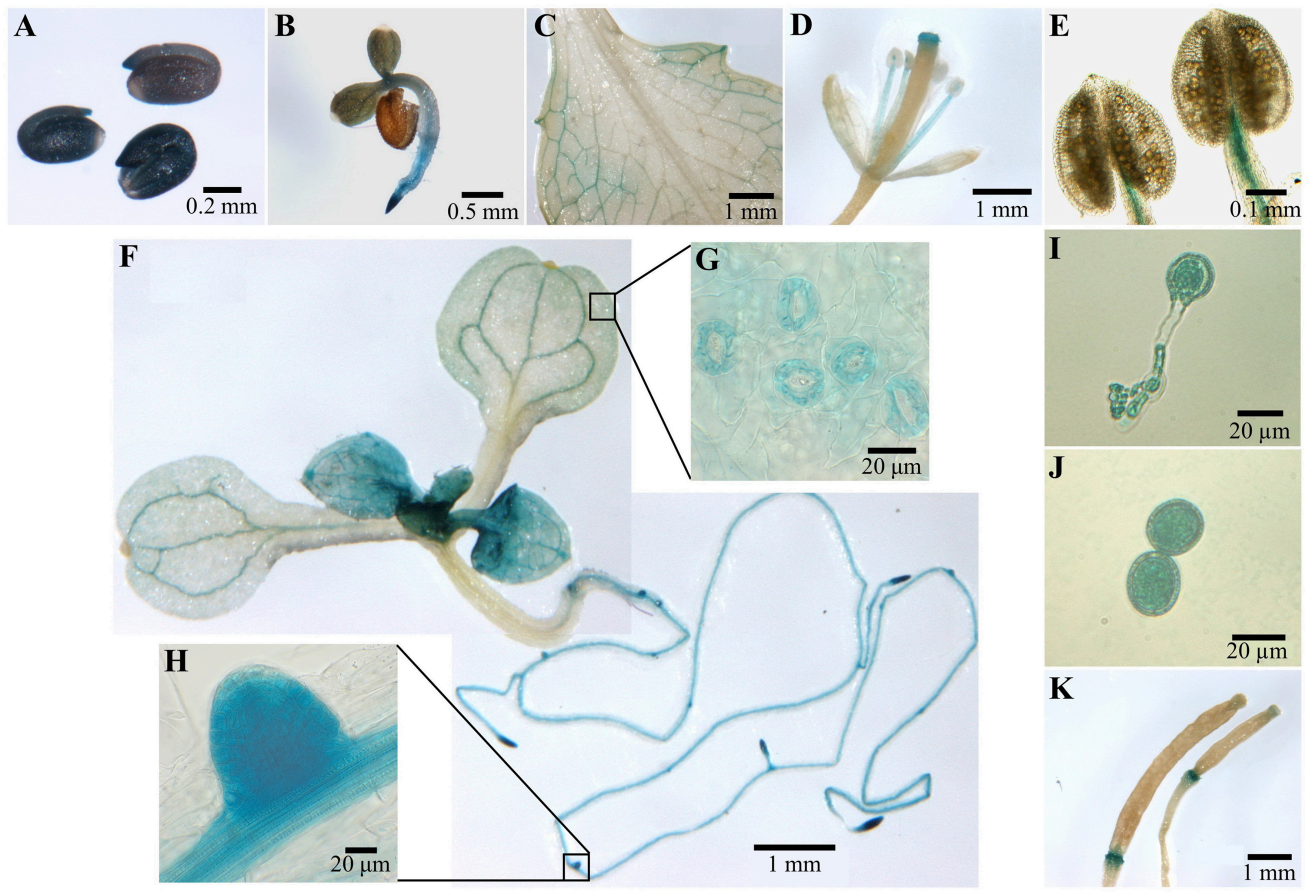
*****COX11:GUS*** promoter analysis**. Plants from three *COX11:GUS* lines (T2 generation) were assayed for GUS activity at various developmental stages by bright-field microscopy. Representative staining patterns are shown. **(A)** Embryos after overnight imbibition and removal of the seed coats. **(B)** A 3-day-old seedling. **(C)** Mature leaf. **(D)** *Arabidopsis* flower. **(E)** Anthers with mature pollen. **(F)** Two images of different parts of a 12-day-old seedling, stitched together. The insert panels show a section of its cotyledon **(G)** and a lateral root primordium **(H)** at a higher magnification. **(I)** Germinated pollen. **(J)** Pollen grain rehydrated in 18% sucrose. **(K)** Siliques at different developmental stages.

In order to determine the tissue expression pattern of *COX11*, genomic DNA comprising about 400 bp upstream and downstream of the putative transcription start site (as annotated in the TAIR data base; Lamesch et al., 2012) was fused with the reporter gene β*-glucuronidase (GUS)* and transformed into the WT background. The downstream region contained almost the entire 5′ UTR including the first intron. Figure 4 summarizes the results of the GUS staining.

After overnight imbibition, a strong promoter activity was observed in the whole embryo (Figure 4A). With the development of the embryo, the *COX11* promoter activity disappeared in most tissues or was below the detection limit of the GUS assay (Figures 4B,F). The GUS activity remained high in meristematic tissues of the shoot and root, as well as in the vascular tissues of source and sink organs (Figures 4C,F,**H**). In contrast, no *COX11* promoter activity was detected in the vascular tissue of transport organs such as the hypocotyl and shoot. These observations agree with reported *COX11* mRNA levels from publicly available microarray data compiled by Genevestigator. For example, *COX11* transcript levels in leaf rosettes are on average approximately two-fold higher than in hypocotyls.

Young leaves near the shoot meristem (Figure 4F) were also stained, indicating strong *COX11* promoter activity. With ongoing maturation, the staining became weaker, except for the vascular tissue and guard cells (Figure 4G). In fully developed leaves (Figure 4C), GUS activity was limited to the vascular tissue close to the leaf periphery. In flowers (Figure 4D), only the stamen vascular tissue and the stigma showed *COX11* promoter activity. Unlike the mature pollen (Figure 4E), the germinated pollen grain exhibited a strong GUS activity (Figure 4I). This was also observed in pollen grains rehydrated in 18% (w/v) sucrose (Figure 4J). In siliques (Figure 4K), the GUS activity was restricted to the abscission zone.

In summary, the *COX11* promoter was mainly active in tissues with high division and/or high metabolic rates. In this regard it resembles the expression patterns of other genes related to COX function, such as COX assembly factor *HCC1* (for homolog of copper chaperone SCO1) (Steinebrunner et al., 2011), the COX subunit *COX5b-1* (Welchen et al., 2004) and the cytochrome *c* encoding genes *CYCT-1* and *CYCT-2* (Welchen and Gonzalez, 2005).

### Knockdown and overexpression of *Arabidopsis COX11*

To further investigate the functions of *Arabidopsis COX11*, we aimed to analyze plant lines with no or with elevated expression of *COX11* [knock-out mutants (KO) and overexpressors (OE), respectively]. Five publicly available T-DNA insertion lines within the *COX11* locus (Supplementary Figure 3) were tested for *COX11* KO. For two of them, Sail_683_B03 and Salk_105793, the presence of the T-DNA insertion could not be confirmed. For the three other lines (Sail_603_G12, Sail_861_D09 and Salk_003445), the T-DNA insertions, all within the *COX11* promoter region, were detected by PCR and homozygous plant lines were selected. For the Sail_861_D09 line, which carries the T-DNA insertion closest to the putative transcription start site, the *COX11* mRNA level was exemplarily determined by qPCR. Unexpectedly, the level was 1.9-fold higher than that of the WT, making this line a weak OE. The *COX11* mRNA levels of the other two lines were not determined, as the sites of the T-DNA insertions in the *COX11* promoter are farther upstream compared with that of the Sail_861_D09 line. Therefore, it can be expected that their impact on *COX11* expression is less pronounced.

To compensate for unavailable KO plant lines, two knock-down lines (KD1 and KD2) were generated by employing the artificial microRNA (amiRNA) technology (see Materials and Methods). Homozygosity of the amiRNA-encoding T-DNA insertion appears to be lethal, as no respective mutant lines could be identified. Therefore, the seedlings had to be cultured on kanamycin to select for heterozygous KD plants. Because these kanamycin-selected seedlings were directly used for qPCR analyses, the influence of kanamycin on the transcript levels of the investigated genes was determined. With the exception of *COPT2* (copper transporter 2) (Supplementary Figure 10), only minor changes were observed. The *COX11* mRNA level was slightly increased (1.19-fold).

**Figure 5:**
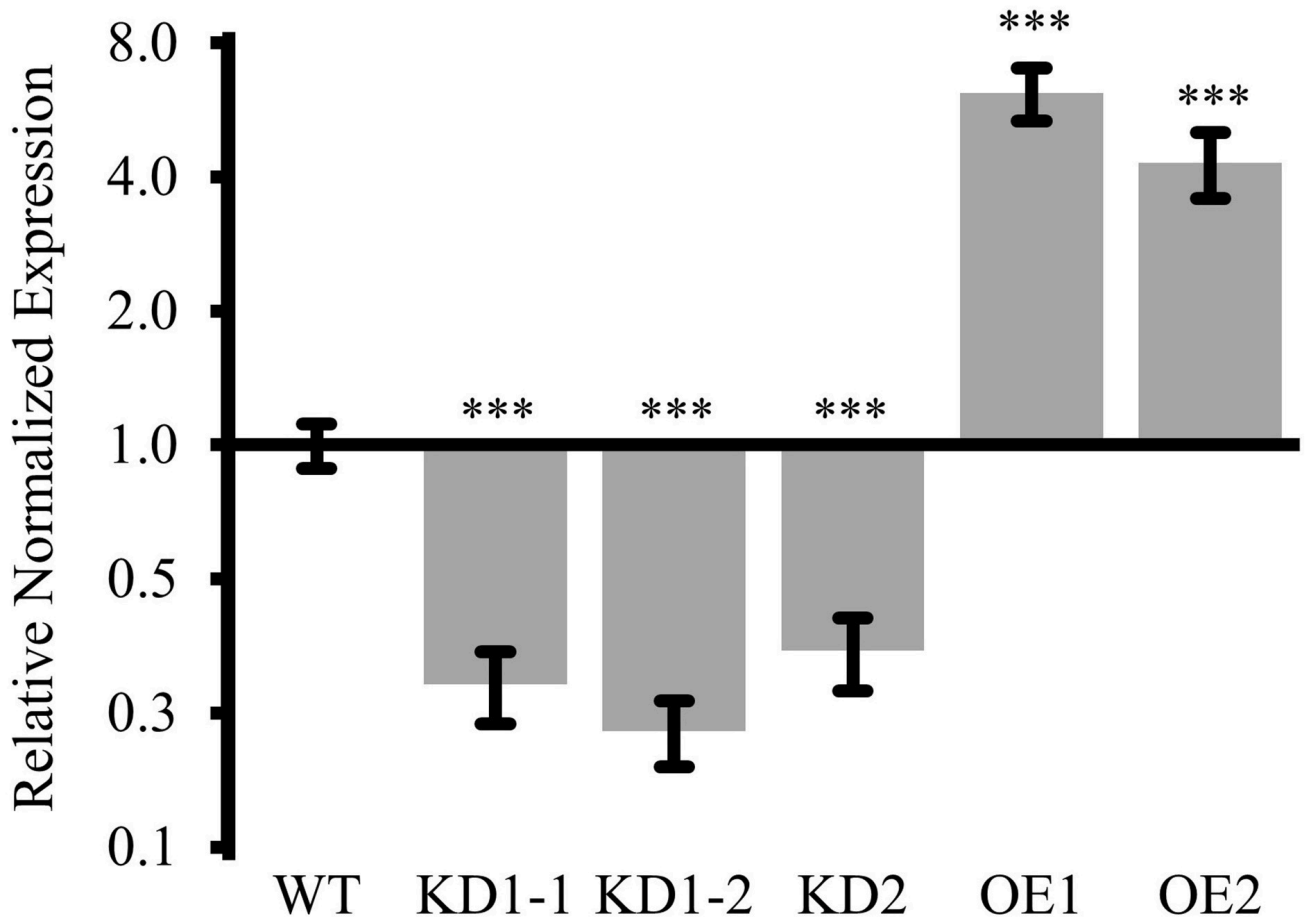
*****COX11*** mRNA levels in KD and OE lines**. *COX11* transcript levels were determined in KD and OE lines by qPCR. RNA was isolated from 14-day-old seedlings cultured on selective or non-selective MS plates. Mean values of mRNA levels in *COX11* mutants were normalized to WT and plotted on logarithmic scale (base two). Values and statistical significance (compared with the WT, ^***^P < 0.001), were calculated with the CFX manager software. Error bars represent ± standard deviation (SD). Individual values are listed in the Supplementary Table 5.

For KD1, seeds were harvested from two individual plants and the respective progeny, KD1-1 and KD1-2, was independently analyzed. In all three lines (KD1-1, KD1-2, and KD2), *COX11* mRNA levels were reduced to 30-40% compared with the WT (Figure 5).

Overexpression was obtained by placing the *COX11* cDNA under the control of the *35S* promoter in the WT background. Two lines, OE1 expressing *COX11* fused to the 3HA-tag sequence, and OE2 expressing untagged *COX11*, were propagated to homozygosity. The two lines showed a six- and four-fold overexpression of *COX11*, respectively, compared with the WT (Figure 5).

### *COX11* knockdown and overexpression affects COX activity

**Figure 6:**
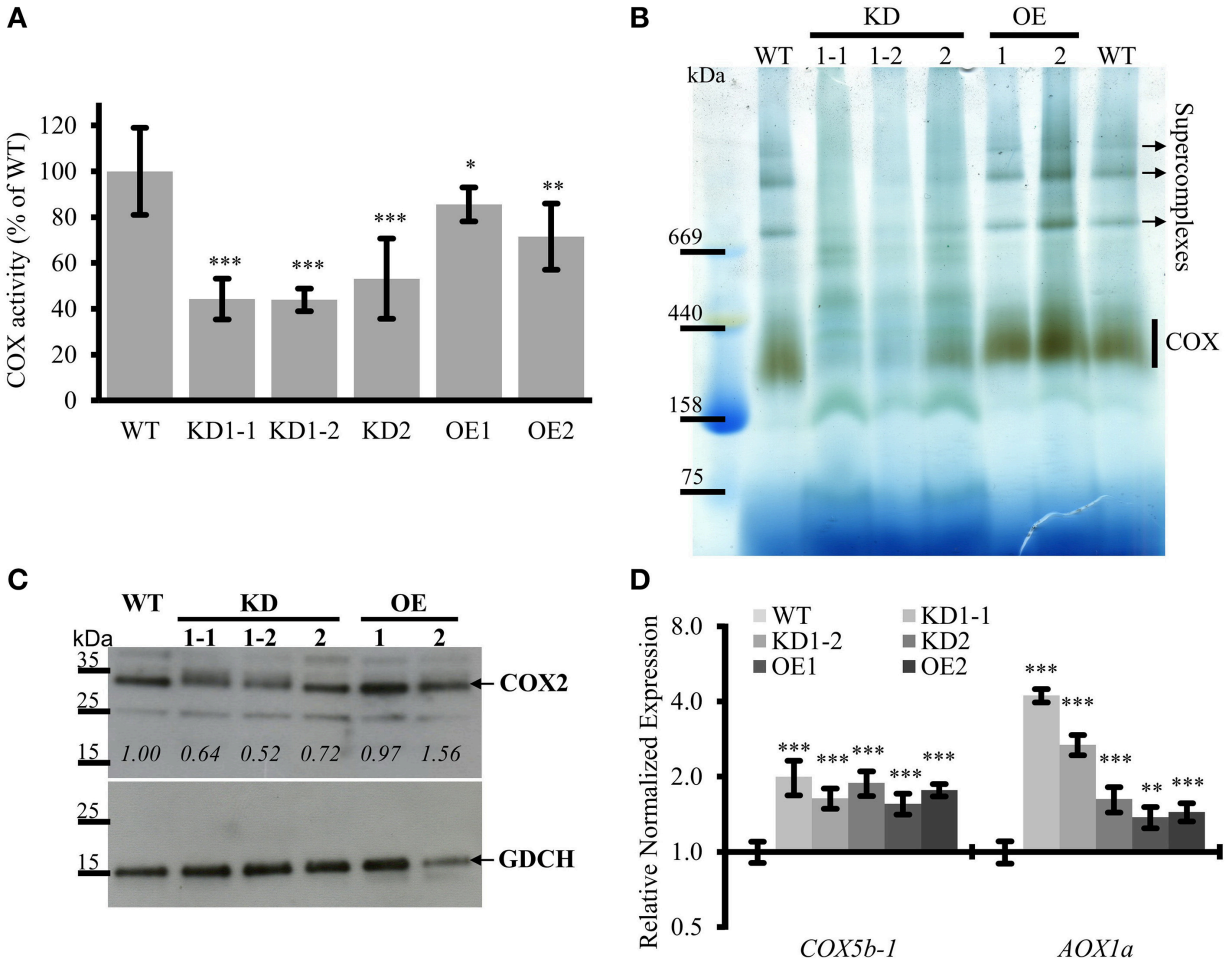
**COX complex activities are reduced in COX11 KD and OE mutants. (A)** COX activities in KD and OE mutants and the WT were measured in three independent mitochondria isolations (each in technical triplicate) and normalized to the WT (= 100%). Asterisks indicate statistical significance calculated with the Student's *t*-test (^*^P < 0.05; ^**^P < 0.01; ^***^P < 0.001). Error bars represent ± SD. **(B)** Mitochondrial complexes from COX11 KD, OE and WT plants were separated by gradient BN-PAGE and subsequently stained in gel for COX complex activity visible as a brown precipitate. For WT, 100 μg of crude mitochondria was loaded, while for the mutants amounts were adjusted (based on citrate synthase activity) to compensate for contaminations. **(C)** Total leaf proteins from WT, KD, and OE plants (6 weeks old) were analyzed by Western blotting, using antibodies against COX2 (theoretical molecular weight of 30 kDa). The membrane was stripped and subsequently incubated with antibodies against GDCH (theoretical molecular weight of 16 kDa) as a loading control. In this experiment T4-generation KD1-1 plants were used (Supplementary Figure 2B). Values in italics indicate estimated COX2 abundance after densitometry analysis and normalization to GDCH amounts. **(D)** qPCR analysis of indicated transcript levels in WT and COX11 mutants. RNA was isolated from 14-day-old seedlings cultured on selective (KD mutants) or non-selective MS (WT) plates. Mean values of mRNA levels in COX11 mutants were normalized to WT and plotted on a logarithmic scale (base two). Values and statistical significance (compared with the WT, ^**^P < 0.01, ^***^P < 0.001) were calculated with the CFX manager software. Error bars represent ± SD. All individual values are listed in Supplementary Table 5.

The main function of COX11 proteins in other organisms is the insertion of copper into the COX complex during its assembly. Therefore, we investigated the effect of *COX11* KD and OE on COX complex activity (Figure 6). Crude mitochondria were prepared from WT, KD and OE plants and assayed for COX activity by measuring the oxidation rate of reduced cytochrome *c*. Citrate synthase activity was used to normalize for the concentration of mitochondrial protein as described previously (Steinebrunner et al., 2014).

The KD lines showed a significant reduction in COX complex activity compared with the WT, about 45% for KD1-1 and KD1-2 and about 55% for KD2 (Figure 6A). Surprisingly, OE lines also exhibited a reduced COX activity (~80% of the WT) (Figure 6A). To confirm the specificity of the COX activity assay, reactions were compared in the presence or absence of the specific COX inhibitor KCN. In the absence of KCN, cytochrome *c* was continuously oxidized. In presence of KCN, this reaction was completely blocked (Supplementary Figure 11A). This result demonstrates that the COX complex activity was responsible for the oxidation of reduced cytochrome *c*.

To corroborate these data, COX complex activity was analyzed by a second method. Crude mitochondria fractions were separated by blue native (BN)-PAGE and subsequently subjected to in-gel COX activity staining (Figure 6B). Brown precipitate marks COX activity, which was the strongest in the size range of 250–400 kDa, corresponding to the COX monomer (230 kDa; Klodmann et al., 2011) and possibly a dimer. Based on the staining intensity, the OE lines and the WT had similar levels of activity, while the KD mutants showed much less COX activity. Especially for the KD1-1 and KD1-2 plants, the activity was so low that it was barely detectable. No brown staining was detected in the presence of KCN, verifying the specificity of the in-gel COX activity staining (Supplementary Figure 11B).

In the upper half of the BN gel, three additional COX activity bands were observed mainly in the WT and OE samples. These bands probably represent respiratory supercomplexes that include the COX complex (Eubel et al., 2004).

To test whether the reduced COX activity associated with *COX11* KD was accompanied by an altered abundance of COX subunits, we analyzed total protein extracts in a Western blot with COX2 antibody (Figure 6C). Densitometric analysis of COX2 abundance, after normalization with GDCH, revealed that COX2 steady-state level was reduced in KD plants, while in the OE lines it was the same or slightly higher compared with the WT. The reduction in KD plants is likely to result from degradation of unassembled respiratory complex subunits as has been reported in yeast and humans (Nakai et al., 1994; Banting and Glerum, 2006; Kovárová et al., 2012). In contrast, at the mRNA level, COX complex subunits were upregulated in both KD and OE plants, as shown exemplarily for *COX5b-1* (Figure 6D).

Previously, it was demonstrated, that in response to respiratory deficiency the gene encoding alternative oxidase 1a (AOX1a) was significantly upregulated (Yuan and Liu, 2012; Yang et al., 2014). In order to test whether this holds also true for the *COX11* KD lines, we determined their *AOX1a* mRNA levels (Figure 6D). We confirmed a negative correlation with the COX activity data, as the KD1-1 and KD1-2 lines exhibited a significantly higher increase of *AOX1a* mRNA than the KD2 line (3–4 vs. 1.6-fold). A similar slight increase of 1.4 was also observed in the OE lines.

### Impaired root growth in *COX11* knock-down and overexpression mutants

**Figure 7:**
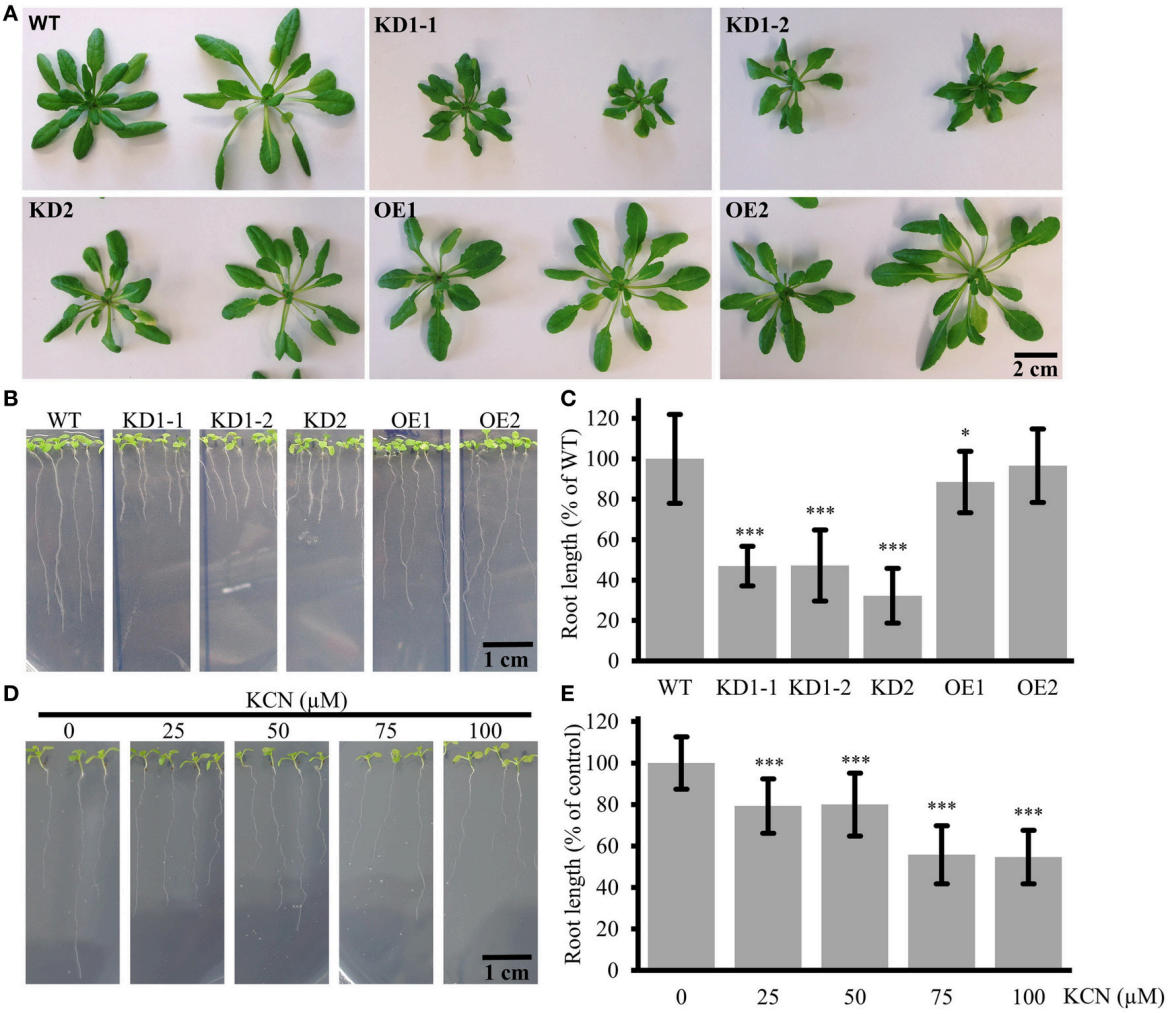
**Leaf and root phenotype of ***COX11*** KD and OE mutants. (A)** Rosettes from 7-week-old plants cultured under short-day conditions. In this experiment T4-generation KD1-1 plants were used (Supplementary Figure 2B). The scale bar applies to all panels. **(B)** Roots of 12-day-old seedlings. The scale bar applies to all panels **(C)** Root lengths from three independent experiments were measured and normalized to the WT (= 100%). Error bars represent ± SD for *n* = 30 (WT, KD1-1, KD1-2, OE1, OE2), 16 (KD2). Asterisks indicate statistical significance calculated with the Student's *t*-test (^*^P < 0.05; ^***^P < 0.001) **(D)** WT roots grown in the presence or absence of KCN. The scale bar applies to all panels. **(E)** Root lengths from two independent experiments were measured and normalized to the untreated control (= 100%). Error bars represent ± SD for *n* = 40 (0, 50, 100 μM KCN), 30 (25, 75 μM KCN). Asterisks indicate statistical significance calculated with the Student's *t*-test (^***^P < 0.001). Absolute and normalized root lengths for **(C,E)** are given in Supplementary Table 5.

In order to investigate a possible effect of *COX11* KD and OE on plant development, we studied the phenotype of respective mutants (Figure 7). The rosettes of 7-week-old KD2 and OE mutants did not differ from the WT in size and morphology, whereas those of the KD1-1 and KD1-2 mutants were smaller with slightly curled leaves (Figure 7A). Apparently, the slightly higher COX activity rates in KD2 mutants compared with the KD1 knockdowns are sufficient to maintain WT leaf growth. Next, we analyzed roots, which are mainly dependent on respiration for energy supply. The KD lines displayed a pronounced difference in root length compared with the WT (Figures 7B,C): after 12 days of growth, the roots were about 50% shorter than in the WT. In the OE mutants, a reduction of root length was less obvious. Thus, the root lengths correlated positively with the COX complex activity of the plants.

Further confirmation that the observed reduction in root length is related to diminished COX complex activity came from experiments employing the specific inhibition of COX by KCN. WT seedlings were cultured on plates supplemented with different concentrations of KCN. As shown in Figures 7D,E, root growth was inhibited in a dosage-dependent manner, demonstrating that COX deficiency leads to reduction in root length. KCN treatment phenocopied the *COX11* mutants, arguing in favor of the KD and OE root phenotype resulting from impaired COX activity.

### Environmental copper affects root growth of *COX11* mutants

**Figure 8:**
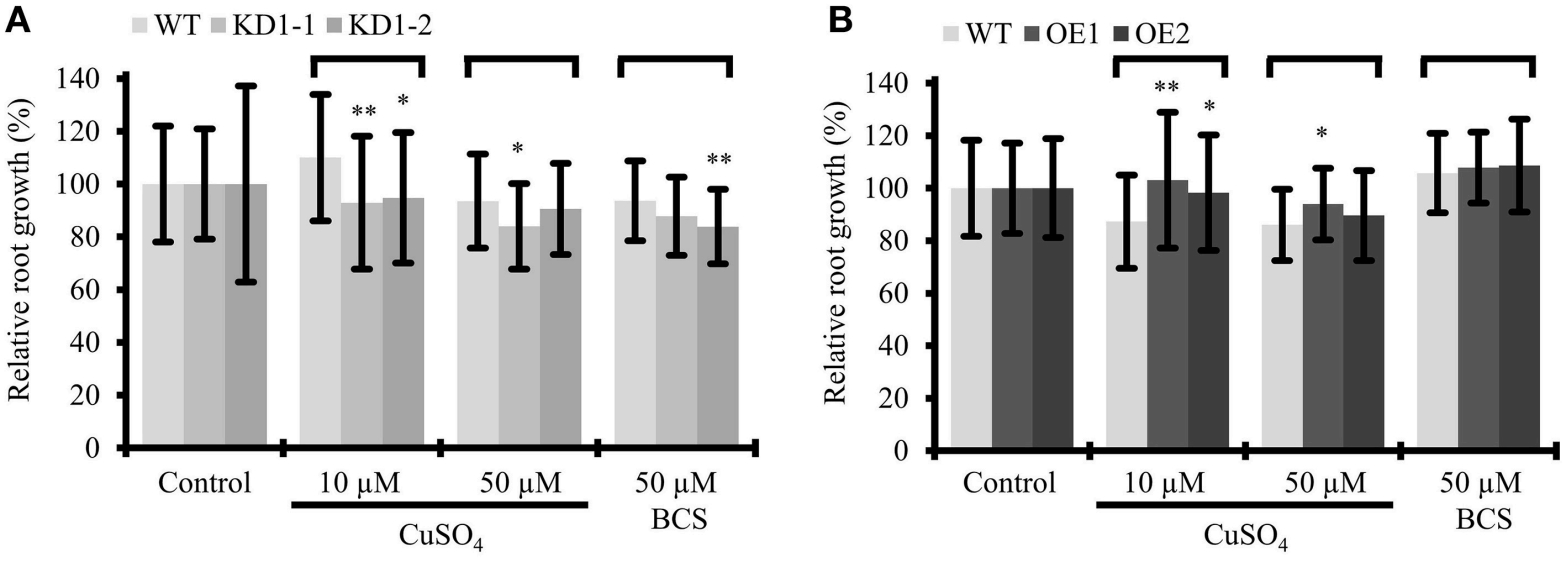
**Influence of Cu treatment on root growth of ***COX11*** mutants**. Relative root growth of 12-day-old WT and KD **(A)** as well as WT and OE **(B)** seedlings cultured on plates with copper excess or deficiency (BCS). Root length is expressed relative to WT or mutant seedlings on control plates. Roots were measured in three independent experiments. Error bars represent ± SD for *n* = ~40 (WT), ~30 (KD1-1, KD1-2), and ~35 (OE1, OE2). Asterisks indicate statistical significance calculated with the Student's *t*-test (mutant line compared with WT, ^*^P < 0.05; ^**^P < 0.01). Individual values are listed in the Supplementary Table 5. BCS, bathocuproinedisulfonic acid.

With the assumption that *Arabidopsis* COX11 is, like other homologs, a copper chaperone, the effect of Cu on root growth was analyzed. WT and *COX11* mutant seedlings were grown together on regular MS plates and on plates supplemented with CuSO_4_ or with the copper chelator BCS (bathocuproinedisulfonic acid). After 12 days, root lengths were evaluated (Figure 8). For more clarity of the figure, the results for the KD2 line are depicted separately in Supplementary Figure 12. Under copper excess, the roots of KD1 plants grew less compared with the WT (Figure 8A). In contrast, the roots of OE grew better compared with the WT (Figure 8B). When seedlings were cultured in the presence of BCS, only the roots of KD1-2 plants grew shorter compared with the WT.

The data are supportive, but not conclusive of COX11 acting as a copper chaperone. Possibly, in the KD lines the reduced amounts of COX11 were unable to efficiently chelate excess copper in the IMS, which in turn exerted its toxic effect and led to reduced root growth. In the OE lines the opposite might have occurred, because the larger amounts of COX11 were able to bind excess copper.

### COX11 knockdown elicits expression changes of genes involved in copper metabolism

**Figure 9:**
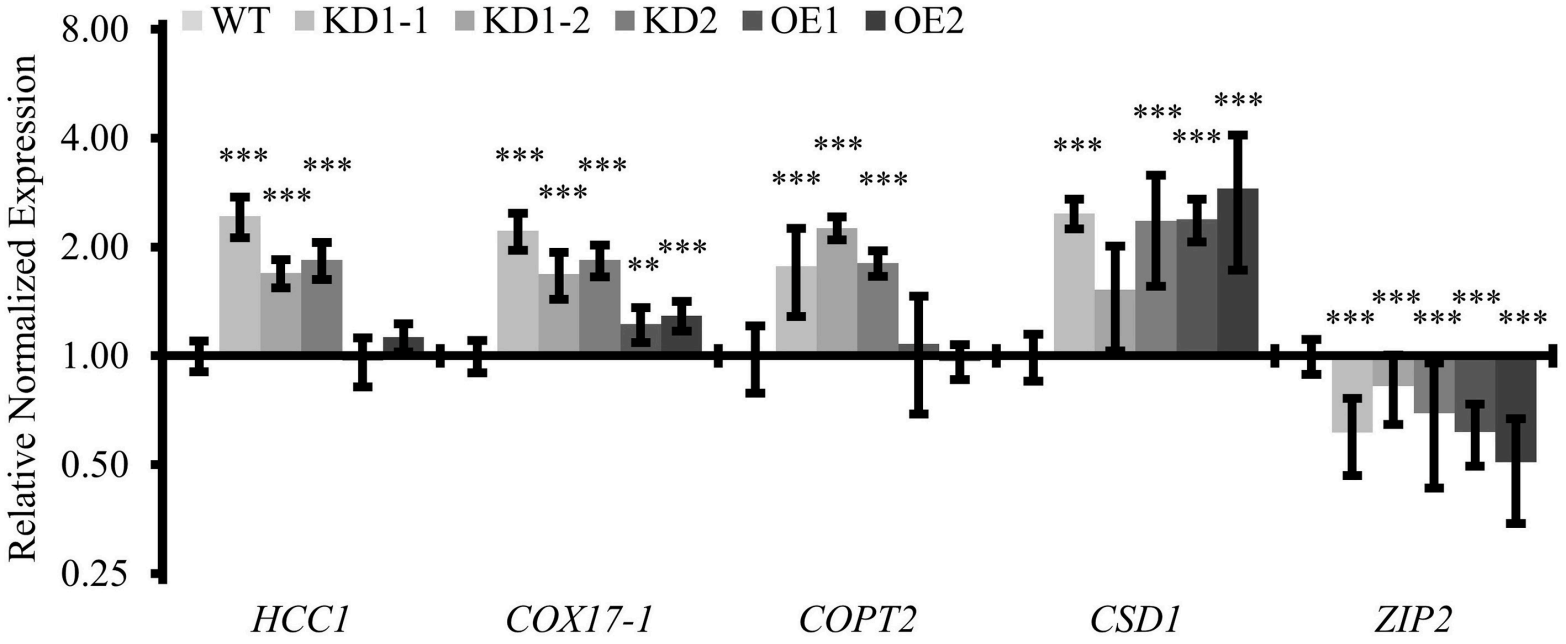
**Quantitative analysis of transcripts associated with copper metabolism**. qPCR analysis of indicated transcripts in WT and *COX11* mutants. RNA was isolated from 14-day-old seedlings cultured on selective (KD) or non-selective MS (WT, OE) plates. Mean values of mRNA levels in *COX11* mutants were normalized to WT and plotted on a logarithmic scale (base two). Values and statistical significance (compared with the WT, ^**^P < 0.01; ^***^P < 0.001) were calculated with the CFX manager software. Error bars represent ± SD. Individual values are listed in the Supplementary Table 5.

Next, we were interested whether the disturbance of COX11 abundance, through KD or OE, affected the regulation of the other mitochondrial copper chaperones HCC1 and COX17-1, both of which have been implicated in COX assembly (Attallah et al., 2007, 2011; Steinebrunner et al., 2011, 2014). This was tested by determining their transcript levels in 14-day-old seedlings by qPCR analysis. Both genes were upregulated about two-fold in the KD lines, but remained almost unaffected or only slightly affected in the OE lines compared with the WT (Figure 9). Possibly this increase, as observed for the subunit *COX5b-1* (Figure 6D), reflects an attempt to counteract the COX deficiency of *COX11* mutants. This result could also indicate a presence of retrograde signaling pathways, which convey mitochondrial dysfunction of COX assembly and Cu metabolism.

Considering this retrograde signaling from mitochondria to the nucleus, we were interested to see whether other genes involved in copper metabolism were also affected in the *COX11* mutant lines. We analyzed the expression levels of *COPT2* (plasma-membrane copper transporter 2), *CSD1* (copper-zinc superoxide dismutase) and *ZIP2* (plasma-membrane metal-ion transporter), all known to be regulated by Cu. We noted a two-fold upregulation of the *COPT2* mRNA level in the KD seedlings, while no difference was observed between the OE and the WT seedlings (Figure 9). For *CSD1* and *ZIP2*, transcript levels were upregulated 2–2.5-fold and downregulated 1.5–2-fold in all lines, respectively. Apparently, the KD of *COX11* had a broad impact on the expression of genes involved in cellular copper homeostasis.

This result prompted us to determine the Cu content in mature leaves of KD mutants. However, as shown in the Supplementary Table 4, it was unaltered in the KD1-1, KD1-2 and OE mutants and even reduced in the KD2 line compared with the WT. An explanation could be that increased COPT2-mediated Cu uptake was limited to seedlings, where we observed the upregulation, or to other tissues than leaves. Indeed, roots for example have 2.4-fold higher *COPT2* mRNA levels than leaves (average microarray data from Genevestigator).

### *COX11* KD and OE leads to reduced pollen germination

**Figure 10:**
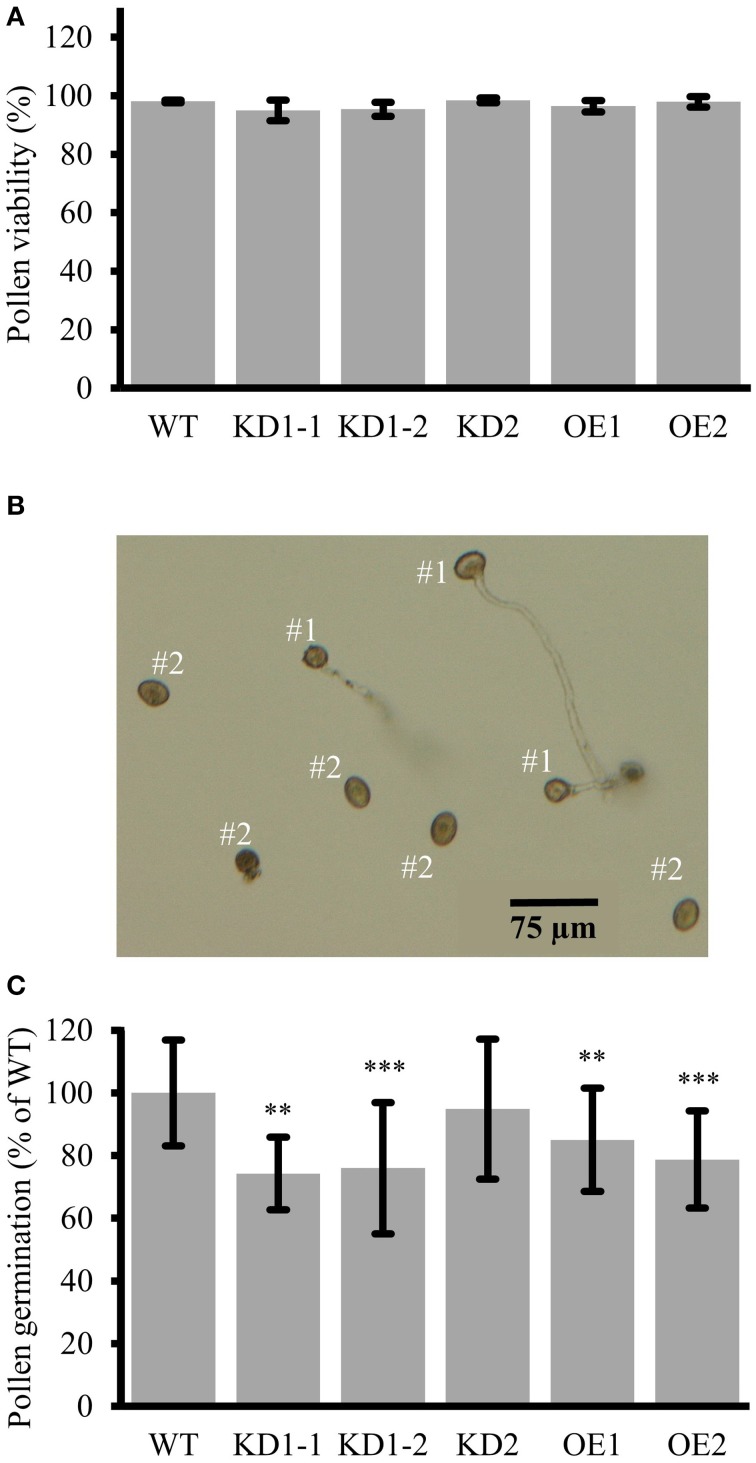
**Reduced pollen germination in ***COX11*** KD and OE mutants. (A)** Pollen viability in *COX11* mutants compared with the WT. Error bars represent ± SD of means from four experiments. Between 400 and 600 pollen grains per plant line were evaluated **(B)** Definition of germinated (#1) vs. non-germinated (#2) pollen grains used for determining germination rates. **(C)** Pollen germination rates of KD and OE mutants were normalized to the WT (= 100%). Error bars represent ± SD of means from 10 experiments. For each experiment, germination of 100–300 pollen grains was evaluated in duplicates or triplicates. Asterisks indicate statistical significance calculated with the Student's *t*-test (^**^P < 0.01; ^***^P < 0.001). Exact values are given in Supplementary Table 5.

It was previously reported that knockdown of rice *COX11* by RNA interference, specifically induced in the tapetum, was linked to defective pollen maturation (Luo et al., 2013). The authors proposed that COX11 has a secondary role in controlling the timing of the oxidative burst by reactive oxygen species (ROS) scavenging. This oxidative signal is as a key signal for the degeneration of the anther tapetum and pollen maturation. In order to test whether the same holds true for *Arabidopsis*, pollen viability of *COX11* mutants was checked by staining with fluorescein diacetate (FDA). Contrary to the situation in rice, more than 95% of the pollen grains in all lines proved to be viable (Figure 10A). However, we cannot rule out the possibility that our approach to generate a *35S*-promoter-driven KD of *COX11* was not effective enough to interfere with pollen viability.

As we observed a substantial *COX11* promoter activity in germinating pollen (Figure 4I), in the next step we analyzed the *in vitro* germination rates of KD and OE pollen. Pollen grains with extended tubes (Figure 10B, #1) were counted as germinated, while pollen grains without any or only small protrusions as non-germinated (Figure 10B, #2). Pollen germination rates were significantly reduced by about 20 and 30%, respectively, in the OE and KD lines compared with the WT, except for the KD2 line (Figure 10C). We speculate that this outlier was due to a loss of the KD effect in the pollen grains, as we observed that *COX11* transcripts had returned to WT levels in the next generation (Supplementary Figure 2A). We also attempted to phenocopy the pollen germination reduction in KD and OE lines by germination of WT pollen in the presence of KCN. This specific COX complex inhibitor led to small reduction of germination rates (Supplementary Figure 13). Addition of 100 μM KCN caused a reduction of about 30% in the germination rate of WT pollen. In summary, COX11 did not impair the pollen viability, but affected pollen germination.

## Discussion

Several members of the COX11 protein family were shown to play a key role in Cu delivery to the COX subunit 1 during COX assembly (Hiser et al., 2000; Carr et al., 2002). In this work, we aimed to investigate if the *Arabidopsis* homolog exerts the same function.

Surprisingly, despite the high sequence and structural similarities *Arabidopsis* COX11 could not complement the respiratory deficiency of yeast Δ*cox11* strain (Figure 1B). However, one has to keep in mind that COX assembly is a very complex process that requires strict spatial and temporal coordination of many proteins, including COX11 (reviewed in Soto et al., 2012). It is possible that specific protein-protein interactions have evolved differently in yeast and *Arabidopsis* mitochondria, preventing the functionality of *Arabidopsis* COX11 in yeast. The observed sporadic growth of yeast cells expressing *CHYM-1* (Figure 1B) might be caused by secondary mutations in some COX11 partner protein(s), allowing the functionality of CHYM-1. Additionally, it cannot be rigorously excluded that this growth is an artifact, caused by prolonged cultivation. Carr et al. (2005) reported similar results for complementation of yeast Δ*cox11* strain with human COX11 (both full-length and chimeras). Obviously, it is not possible to conclude the function of homologous proteins solely based on sequence similarity.

Subsequently, we were able to show that *Arabidopsis* COX11 protein is localized to mitochondria (Figure 2), where it is anchored in the inner mitochondrial membrane (Figure 3) like the yeast COX11 (Carr et al., 2005; Khalimonchuk et al., 2005). Furthermore, we found that the COX activity was reduced in *COX11* KD and OE plant lines (Figure 6A). This result agrees with a function of *Arabidopsis* COX11 in the assembly the COX complex, as shown for other organisms (Hiser et al., 2000; Carr et al., 2005; Banting and Glerum, 2006). The question arises why overexpression of *COX11* also lowers COX activity. A high concentration of the COX11 protein could lead to its inactivation due to the disturbance of protein stoichiometry between COX11 and its auxiliary factor(s). For example, it was recently reported that in yeast, COX19 is required to maintain COX11 in an active state (Bode et al., 2015). A higher concentration of COX11 could result in a depletion of active COX19. As an alternative explanation, one could envisage the titration of essential factors such as Cu (reviewed in Prelich, 2012). The elevated amount of COX11 possibly competes with HCC1 for Cu loading from COX17, disturbing Cu transfer to COX2, subsequently leading to a reduced COX activity (Figure 6A). These hypotheses explain the phenotypes of OE plants cultured under normal conditions; however, they do not explain why OE plants, when exposed to excess copper grow better compared with the WT (Figure 8B). Possibly, the surplus COX11 in OE plants promotes root growth by sequestering excess Cu, thus alleviating Cu toxicity. This together with the results for KD plants (Figure 8A), where the reduced amount of COX11 probably fails to sequester excess Cu, is in favor of COX11 being a copper binding protein. We conclude that the *Arabidopsis* COX11 homolog is essential for assembly of the COX complex, possibly by inserting Cu into the COX1 subunit, as shown for other organisms (Hiser et al., 2000; Banting and Glerum, 2006; Thompson et al., 2010).

Support for a role of COX11 in energy metabolism is provided by its expression pattern (Figure 4). The *COX11* promoter was predominantly active in tissues with a high-energy demand, e.g., in meristems to sustain high division rates or in vascular tissues of leaves and roots to maintain phloem loading and unloading, respectively (Taiz and Zeiger, 2010). The promoter activity was also high in imbibed embryos, which need to repair and differentiate their mitochondria for ATP production prior to germination (reviewed in Weitbrecht et al., 2011). All these tissues depend on the continuous formation of respiratory chain complexes and hence require sufficient amounts of assembly factors like COX11.

In the course of our study, we observed an upregulation of several genes both in *COX11* KD and OE plants (Figures 6D, 9). The higher expression levels of COX subunits and assembly factors (*COX5b-1, HCC1*, and *COX17-1*) in the KD lines may reflect a cellular reaction to compensate for the reduced COX complex activity, by elevating the formation of COX subunits and the factors necessary for their assembly. Probably due to a less pronounced COX deficiency in OE lines, only the level of *COX5b-1* mRNA was significantly elevated. The upregulation of the expression of the *AOX1a* gene, both in KD and OE plants, may indicate a further counter-reaction of the cells against COX deficiency. Remarkably, in the KD lines the expression of the *COPT2* gene was also upregulated, which can possibly be regarded as an attempt of the cells to increase the cellular concentration of Cu to allow COX complex assembly. If indeed, the KD mutants were Cu deficient, their *CSD1* levels should be lower and *ZIP*2 levels higher (Wintz et al., 2003) compared with the WT. However, both genes were regulated in the exact opposite way (Figure 9). Therefore, it seems unlikely that increased *COPT2* amounts in KD plants are related to Cu deficiency. Instead, the high number of stress-and ROS-responsive regulatory elements in the promoter region of *COPT2* (Peñarrubia et al., 2015) suggests that *COPT2* might be upregulated by stress conditions due to impaired COX function. In agreement with this, *CSD1*, a known ROS detoxifier, is upregulated in KD mutants (Figure 9).

Both the knockdown and overexpression of *COX11* interferes with the protein's function and has substantial phenotypic consequences. The most striking effect was the root growth inhibition in *COX11* KD plants (Figures 7B,C). The question arises whether energy deficiency due to reduced COX activity or other effects of COX11 disturbance are the underlying reason(s) for reduced root growth. The shorter-root phenotype positively correlated with a diminished COX activity (Figure 6A). Importantly, the same phenotype could be mimicked by treatment of WT seedlings with KCN (Figures 7D,E), which specifically inhibits COX. Therefore, it seems probable that the energy deficit caused by the lower COX activity results in root growth inhibition in *COX11* KD and OE plants. Similar root shortening was observed in other *Arabidopsis* mutants with impaired energy production due to deficient respiratory chain complexes (Yuan and Liu, 2012; Huang et al., 2013; Yang et al., 2014).

A more subtle, but still noticeable phenotypic change in KD mutants was curling of the leaf blades (Figure 7A). It was more pronounced in the KD1 lines, in which *COX11* expression was more strongly suppressed than in the KD2 line (Figure 5). This leaf phenotype was found to be associated with mitochondrial dysfunction, and can be explained by the high demand of energy required for morphogenetic processes, such as cell division (Van Aken et al., 2007; Gibala et al., 2009).

Previously, it was suggested that COX11 is involved in pollen maturation, because silencing of *COX11* in rice produced non-viable pollen (Luo et al., 2013). Furthermore, near-complete COX-deficient *Arabidopsis* mutants were found to produce non-viable pollen as well (Dahan et al., 2014). However, pollen viability was not affected in the *COX11* mutant lines of our study (Figure 10A), possibly because the knockdown efficiency was not strong enough. Instead, we observed that pollen germination was reduced, in both the KD and OE lines (Figure 10C). Again it is likely that this effect is at least partly due to the reduced COX activity, as KCN-induced COX deficiency also affected pollen germination (Supplementary Figure 13). However, this effect was significantly less pronounced than in case of root growth (Figure 7E). Additionally, the KD and OE pollen germination rates did not correlate well with the respective COX activities (Figures 6A, 10C). In a previous report, it was shown that petunia pollen germination does not rely on respiration and that aerobic glycolysis can provide sufficient energy for this process (Gass et al., 2005). Therefore, *Arabidopsis* COX11 may contribute to pollen germination not only via its function in energy supply, but also through unrelated function(s). A speculative fitting role could be in oxidative signaling. As shown by Speranza et al. (2012), a tightly regulated ROS appearance is essential for pollen activation and pollen tube emergence. *Arabidopsis* COX11 could participate in the ROS metabolism regulation as suggested for the COX11 homologs in rice (Luo et al., 2013) and yeast (Banting and Glerum, 2006; Khalimonchuk et al., 2007). Consequently, its knockdown or overexpression could disturb the ROS signaling and thus germination.

In summary, the characterization of *COX11* KD and OE mutants clearly documents the essential role of *Arabidopsis* COX11 for COX activity, and that this function strongly affects plant development and performance. Additionally, our data hint at an additional function of *Arabidopsis* COX11 in ROS signaling.

## Author contributions

IR, GR, and IS conceived and designed the research. IR performed the research. NM designed and generated the KD lines. IR analyzed the data, with IS co-analyzing root growth and imaging data. IR, GR, and IS wrote the article.

## Funding

This work was supported by a PhD fellowship to IR from the Dresden International Graduate School for Biomedicine and Bioengineering (DIGS-BB), which is funded by the DFG (German Research Foundation). The grant STE 1455/5-1 from DFG awarded to IS supported a research stay of IR at the Universidad Nacional del Litoral in Santa Fe, Argentina, which included training in methods employed in this work. The publication fee for this article was covered by the DFG grant RO 1299/9-3.

### Conflict of interest statement

The authors declare that the research was conducted in the absence of any commercial or financial relationships that could be construed as a potential conflict of interest.
