# Supplementary material for: The Arabidopsis COX11 Homolog is Essential for Cytochrome c Oxidase Activity
Source: Front Plant Sci. 2015 Dec 18;6:1091. doi: 10.3389/fpls.2015.01091 (PMC4683207; doi:10.3389/fpls.2015.01091)
Supplement: Supplementary file 1 [file Table1.PDF]

1.2. Supplementary Tables

SUPPLEMENTARY TABLE 1 | Cloning and screening primers used in this study.

Name	Sequence (5’ → 3’)
Cloning primers[1]	
cox11miR-s	GATGAGAGCATAAAATAGTCCTTTCTCTCTTTTGTATTCC
cox11miR-a	GAAAGGACTATTTTATGCTCTCATCAAAGAGAATCAATGA
cox11miR*s	GAAAAGACTATTTTAAGCTCTCTTCACAGGTCGTGATATG
cox11miR*a	GAAGAGAGCTTAAAATAGTCTTTTCTACATATATATTCCT
NB47F	CATTTCATTTGGAGAGAACACG
NB47R[2]	CGAAACCGATGATACGAACG
AtcDNA B1F	GGGGACAAGTTTGTACAAAAAAGCAGGC TACCATGTCGTGGTCGAAAGCTTG
AtcDNA B2RFus	GGGGACCACTTTGTACAAGAAAGCTGGGTCTCCATTGGTTTCTTGAACTGG
SccDNA B1F	GGGGACAAGTTTGTACAAAAAAGCAGGCTACCATGATAAGAATATGTCCC
SccDNA B2R	GGGGACCACTTTGTACAAGAAAGCTGGGTTTAATTTGAGTTGTCTTTCC
At(N) OL R	CACCAAAACCAGTACGAGCACAAATGGCTCTATAGAGTGGCACAGCAGCGTAAGTTAACC
Sc(C) OL F	TGTTGTCTTTGGTATGGTGGGGTTAACTTACGCTGCTGTGCCACTCTATAGAGCCATTTG
Sc(N) OL R	CTCCATATCCAGTAGCTTGGCAGAATGTTCTATACAATGGTACCGCTGCATATGCCAAACC
At(C) OL F	CGTCGCGGTACTTTTCTTGGGTTTGGCATATGCAGCGGTACCATTGTATAGAACATTCTGC
AtPr B1F	GGGGACAAGTTTGTACAAAAAAGCAGGCTAATGTTTTGGGCTTAGACTTG
AtPr B2R	GGGGACCACTTTGTACAAGAAAGCTGGGTGAAATTATTTTAATCTATCGACAC
AtRFP B1F	GGGGACAAGTTTGTACAAAAAAGCAGGCTACCATGGCCTCCTCCGAGGAC
AtRFP B2R	GGGGACCACTTTGTACAAGAAAGCTGGGTTTAGGCGCCGGTGGAGTG
T-DNA insertion screening primers	
Cox11 5UTR F[3]	GTGTCTGTGTCTCACTGTGTCG
Cox11exon5big R[3,4]	CTTCATGGGAGTGACATTGTATGTCGAGAC
AtCox11 5UTR R[3]	GGGAGGGAGATCAAAGAC
AtCox11 genF2[3]	GAGGATCACGATAGCCATGAGG
AtCox11 int1 R[3]	CCAATTTCTCCCTTCCGCAACC
Left B F[3]	GCATCTGAATTTCATAACCAATCTCGATACAC
pROK2 LBb2[3]	GCGTGGACCGCTTGCTGCAACT
35S-1b[4]	GCTCCTACAAATGCCATCATTGC
RFP R[4]	CCTTGGTCACCTTCAGCTTG
CHEQAMICOX11[2]	AAGGACTATTTTATGCTCTCA

[1]Underlined regions of the primers recognize the target site.
[2] Primers used for confirmation of the genomic insertion of the amiRNA construct.
[3] Primers used for detection of T-DNA insertions in SAIL and SALK mutant lines.
[4] Primers used for confirmation of cloned constructs carrying T-DNA insertions.
