# Supplementary material for: The Arabidopsis COX11 Homolog is Essential for Cytochrome c Oxidase Activity
Source: Front Plant Sci. 2015 Dec 18;6:1091. doi: 10.3389/fpls.2015.01091 (PMC4683207; doi:10.3389/fpls.2015.01091)
Supplement: Supplementary file 2 [file Table2.PDF]

SUPPLEMENTARY TABLE 2 | Primers used for qPCR.

Locus	Primer Name	Primer Sequence (5' → 3')	Conc. each (µM)	Efficiency (%)	Amplicon Size (bp)		Comment	Reference
					cDNA	gDNA		
At1g02410	*COX11* F<br>*COX11* R	GATTGACATGCCGGTCTTCT<br>TGGTTTCTTGAACTGGAACAGA	0.33	102.6	164	N/A	spans exon/exon	this work
At1g13320	*PP2A* F<br>*PP2A* R	CCTGCGGTAATAACTGCATCT<br>CTTCACTTAGCTCCACCAAGCA	0.33	95.5	142	356	spans 2 introns	(Czechowski et al., 2005)
At3g18780	*ACT2* F<br>*ACT2* R	GGTAACATTGTGCTCAGTGGTGG<br>AACGACCTTAATCTTCATGCTGC	0.33	100.5	108	108	spans no introns	(Czechowski et al., 2005)
At3g22370	*AOX1a* F<br>*AOX1a* R	GGAGGCTTCCTGCTGATGCGACA<br>AGCTGGAGCTTCCTTTAGTTCACGACC	0.16	102.7	134	214	spans 1 intron	this work
At3g08950	*HCC1* F<br>*HCC1* R	GGCCCGATCTTACCGGGTTT<br>CAACGCCGTCTGTCAACGAG	0.25	99.8	158	391	spans 1 intron	this work
At3g46900	*COPT2* F<br>*COPT2* R	TTGGGGTAAGAACACGGAGGT<br>TGACACGTAGGATCGGTGAATG	0.5	96.1	137	137	gene has no introns	(Del Pozo et al., 2010)
At3g15352	*COX17-1* F<br>*COX17-1* R	CTGATCAGCCAGCACAAAATGGAT<br>CTCAGCTTCTTGGTATCAGGGC	0.12	95.3	127	127	gene has no introns	this work
At3g15640	*COX5b-1* F<br>*COX5b-1* R	GACAAGCGAATTGTGGGCTG<br>AGTACTGAGTGCAAACCGGG	0.25	104.3	112	238	spans 1 intron	this work
At1g08830	*CSD1 F*<br>*CSD1 R*	CCCTGAGGATGCTAATCGACAT<br>TGGCAATCAGTGATTGTGAAGG	0.5	92.9	90	N/A	Spans 1 intron and exon/exon	(Del Pozo et al., 2010)

At5g59520	*ZIP2 F*<br>*ZIP2 R*	ACGTTGCGGTTAACCATCTC<br>CGAGGAAGACGGCAATAAAC	0.5	85.7	96	96	(Del Pozo et al., 2010)

The optimal final primer concentrations (conc.) and primer pair efficiencies were experimentally determined. The amplicon size of the cDNA and of the potential contamination genomic DNA (gDNA) are given. F = forward, R = reverse, N/A = not applicable.

Czechowski, T., Stitt, M., Altmann, T., Udvardi, M.K., Scheible, W.R.R. (2005). Genome-wide identification and testing of superior reference genes for transcript normalization in *Arabidopsis*. *Plant Physiol.* 139: 5-17. doi: 10.1104/pp.105.063743

Del Pozo, T., Cambiazo, V., González, M. (2010). Gene expression profiling analysis of copper homeostasis in *Arabidopsis thaliana*. *Biochem. Biophys. Res. Commun.* 393: 248-252. doi: 10.1016/j.bbrc.2010.01.111
