# Supplementary material for: The Arabidopsis COX11 Homolog is Essential for Cytochrome c Oxidase Activity
Source: Front Plant Sci. 2015 Dec 18;6:1091. doi: 10.3389/fpls.2015.01091 (PMC4683207; doi:10.3389/fpls.2015.01091)
Supplement: Supplementary file 3 [file Table3.PDF]

SUPPLEMENTARY TABLE 3 | Summary of different colocalization coefficients.

	Colocalization coefficient (± SD)		
	Pearson's coefficient	Mander's coefficient 1	Mander's coefficient 2
mt-GFP + mRFP	0.61 ± 0.13	0.93 ± 0.08	0.20 ± 0.13
mt-GFP + COX11-mRFP	0.87 ± 0.05	0.86 ± 0.06	0.82 ± 0.08

Coefficients were calculated with the JACoP plugin for Fiji from a series of confocal images of different cells from both plant lines. The Pearson's correlation coefficient estimates the spread of the pixel distribution in regard to a fitted line in scatter plots (**Supplementary Figure 8**), and its values range between -1 and 1, with 1 indicating complete colocalization (Bolte and Cordelières, 2006). Mander's overlap coefficient 1 is defined as a ratio of the number of green pixels with the value for the red channel above zero and the number of total green pixels. Mander's coefficient 2 = Mander's coefficient 1, but for the red channel (Manders et al., 1992).

Bolte, S., Cordelières, F.P. (2006). A guided tour into subcellular colocalization analysis in light microscopy. *J. Microsc. (Oxford, UK)* 224: 213-232. doi: 10.1111/j.1365-2818.2006.01706.x

Manders, E.M.M., Stap, J., Brakenhoff, G.J., Van Driel, R., Aten, A. (1992). Dynamics of three-dimensional replication patterns during the S-phase, analysed by double labelling of DNA and confocal microscopy. *J. Cell Sci.* 103: 857-862.
