# Supplementary material for: The Arabidopsis COX11 Homolog is Essential for Cytochrome c Oxidase Activity
Source: Front Plant Sci. 2015 Dec 18;6:1091. doi: 10.3389/fpls.2015.01091 (PMC4683207; doi:10.3389/fpls.2015.01091)
Supplement: Supplementary file 4 [file Table4.PDF]

SUPPLEMENTARY TABLE 4 | Copper content in *COX11* KD, OE and WT plants.

WT	KD1-1	KD1-2	KD2	OE1	OE2
8.3 ± 0.3	8.3 ± 1.0	9.0 ± 1.2	6.1 ± 0.2***	7.9 ± 0.6	7.2 ± 0.7

Presented values (± SD) indicate the copper content in leaves. The copper amounts are expressed as mg of copper per kg of dry leaf weight (mg kg^{-1}). In this experiment T4-generation KD1-1 plants were used (**Supplementary Figure 2B**). All values are averages of triplicate measurements. Asterisks indicate statistical significance compared with the WT (Student's *t* test). ***$P < 0.001$.
