# Supplementary material for: The Arabidopsis COX11 Homolog is Essential for Cytochrome c Oxidase Activity
Source: Front Plant Sci. 2015 Dec 18;6:1091. doi: 10.3389/fpls.2015.01091 (PMC4683207; doi:10.3389/fpls.2015.01091)
Supplement: Supplementary file 6 [file Image1.PDF]

Supplementary Material

The Arabidopsis COX11 homolog is essential for cytochrome *c* oxidase activity

Ivan Radin, Natanael Mansilla, Gerhard Rödel*, Iris Steinebrunner

*** Correspondence:** Gerhard Rödel: gerhard.roedel@tu-dresden.de

1. Supplementary Figures and Tables

1.1. Supplementary Figures

A

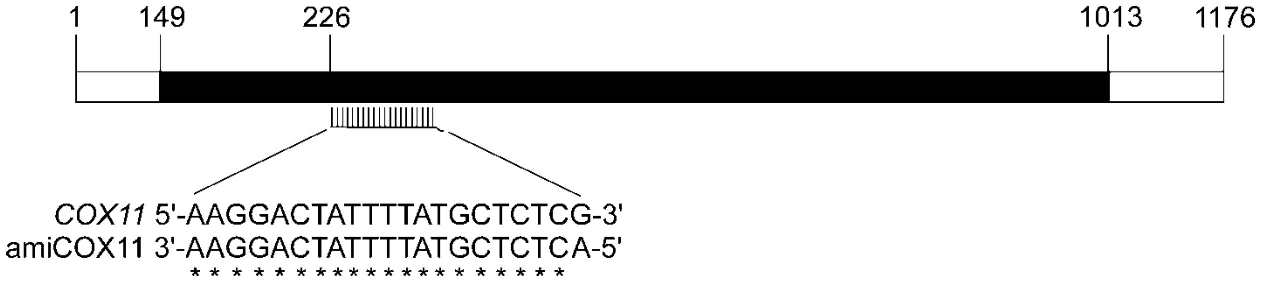

B

GGATCCTAGGAATATATATGTAGAAAAGACTATTTTAAGCTCTCTTCACAGGTCGTGATATGATT
CAATTAGCTTCCGACTCATTCATCCAAATACCGAGTCGCCAAAATTCAAACTAGACTCGTTAAAT
GAATGAATGATGCGGTAGACAAATTGGATCATTGATTCTCTTGATGAGAGCATAAAATAGTCCTT
TCTCTCTTTTGTATTCCCTGCAG

SUPPLEMENTARY FIGURE 1│Design of amiRNA for *COX11* silencing. (A) Schematic model of the amiRNA targeting site for *COX11* silencing. The *COX11* mRNA sequence is depicted schematically, with white boxes representing the UTRs and the black box designating the coding sequence. The numbers indicate the first and final nucleotide of the mRNA sequence as well as the start nucleotide of the coding sequence, the 3' UTR and the amiRNA recognition site. The reverse complementary sequence of the amiRNA matching the *COX11* transcript is marked by asterisks. (B) Sequence of the *COX11*-targeting amiRNA cloned into the pNB47 vector. Used restriction sites are single (*Bam*HI) or double (*Pst*I) underlined, the dashed line indicates the amiRNA.
