# Supplementary material for: The Arabidopsis COX11 Homolog is Essential for Cytochrome c Oxidase Activity
Source: Front Plant Sci. 2015 Dec 18;6:1091. doi: 10.3389/fpls.2015.01091 (PMC4683207; doi:10.3389/fpls.2015.01091)
Supplement: Supplementary file 7 [file Image2.PDF]

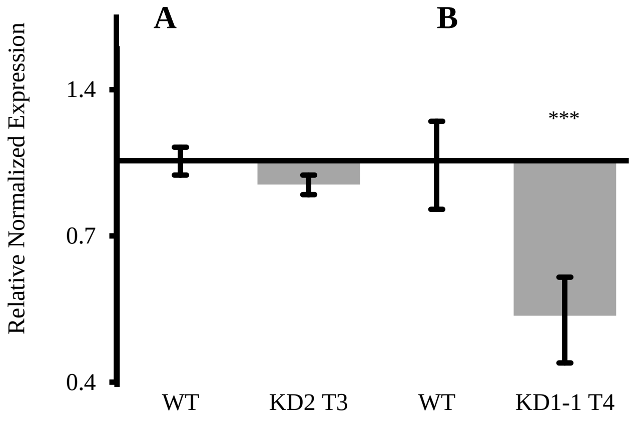

SUPPLEMENTARY FIGURE 2 | *COX11* mRNA levels in the T3 generation of KD2 plants and the T4 generation of KD1-1 plants. (A) For determination of *COX11* transcript amounts in T3-generation KD2 plants, technical triplicates were used. The experiment revealed that, in KD2 plants, the KD effect was not carried over to the next generation. **(B)** For T4-generation KD1-1 plants, *COX11* transcript amounts were determined with biological and technical triplicates. The T3-generation KD1-1 plants retained similar KD levels as observed for the T3 generation (**Figure 5**). RNA was isolated from 14-day-old seedlings cultured on selective (KD mutants) or non-selective (WT) MS plates. Mean values of mRNA levels in *COX11* mutants were normalized to WT and plotted on a logarithmic scale (base two). Values and statistical significance (compared with the WT, ***$P < 0.001$) were calculated with the CFX manager software. Error bars represent ± SD. Individual values are listed in the **Supplementary Table 5.**
