# Supplementary material for: The Arabidopsis COX11 Homolog is Essential for Cytochrome c Oxidase Activity
Source: Front Plant Sci. 2015 Dec 18;6:1091. doi: 10.3389/fpls.2015.01091 (PMC4683207; doi:10.3389/fpls.2015.01091)
Supplement: Supplementary file 8 [file Image3.PDF]

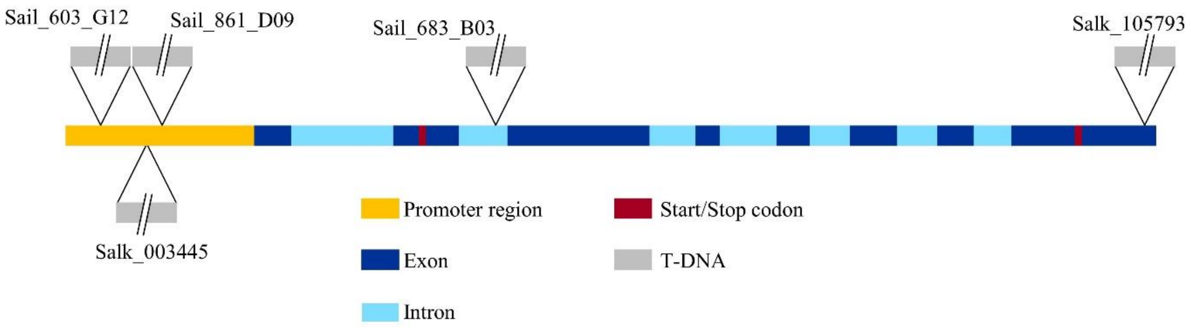

SUPPLEMENTARY FIGURE 3 │ Schematic diagram of *COX11* T-DNA mutations. The *COX11* locus (drawn to scale) and positions of T-DNA insertions in different mutants are depicted. Because of their large sizes, T-DNA insertions are not drawn to scale. The names (from the TAIR database) for each plant T-DNA insertion line are also given.
