# Supplementary material for: The Arabidopsis COX11 Homolog is Essential for Cytochrome c Oxidase Activity
Source: Front Plant Sci. 2015 Dec 18;6:1091. doi: 10.3389/fpls.2015.01091 (PMC4683207; doi:10.3389/fpls.2015.01091)
Supplement: Supplementary file 9 [file Image4.PDF]

```
R.sphaeroides             ------------------------------------------------------------
H.sapiens                 ----------------------------------MGGLWRPGWRCVPFCGWRWIHPGSPT
D.melanogaster            ------------------------------------------------------------
N.crassa                  MSSMPRFVGRRAVAV--QSFSQSASWACFSCRQEFQNQWRRQLSFDT-------------
S.cerevisiae              -------------------------------------MIRICPIVRS-------------
R.prowazekii              ------------------------------------------------------------
D.discoideum              ---MSRLFNNFLLKTNKNQFIQSFSTKSQINNNIFNNLIKNEKISLP-------------
C.reinhardtii             ---------------------MS---------------LRRAA-----------------
R.americana               ------------------------------------------------------------
A.thaliana                ---------------------MS---------------WSKACRGTR-------------
O.sativa                  ---------------------MP----------------------PP-------------

R.sphaeroides             ------------------------------------------------------------
H.sapiens                 RAAERVEPFLRPEWSGTGGAERGLRWLGTW------KRCS---LRAR------HPA----
D.melanogaster            ---------------------------MMR------SLCA---LRGQ------CQQLFRS
N.crassa                  -----EAPSAAS-------SD-------PRR-----KMTT----AAQQQASGAKKQQ---
S.cerevisiae              -----KVPLLGT-------FLRSDSWLAPHALALRRAICKNVALRS--------------
R.prowazekii              ------------------------------------------------------------
D.discoideum              -----PPPFLSS-------SS-------PLSM-------NNI-------FNRNNKNLYTT
C.reinhardtii             -------TLFAR-------SFAESASSLPVEAGVGARLCTGASTSGRQAWPGAAAGVYAR
R.americana               ------------------------------------------------------------
A.thaliana                -----ISSYLEN-------LH--RTSQYPRT-----ILCSRYYTHG---ACKSNEHYLRS
O.sativa                  -----PPPSLAR-------LH--QRLS--LS-----LLRG----RS---PPAAADAFLRR

R.sphaeroides             -----------------------------------------------------MSLSPHQ
H.sapiens                 --------------LQPPRRPKSS----NPFT-----------------RAQEEERRQNK
D.melanogaster            -------------SIRPQNSVHKS----QQFWR----------MKSTDSPEDAARKLRAK
N.crassa                  -----SDRAWR-----WFSTEGASRRQQQQ--QTRS-QSSRTGVSPEMERVRAEYKKRNQ
S.cerevisiae              ---------YSVNSEQ----PK------HTFDISKL--TRNEIQQLRELKRARERKFKDR
R.prowazekii              -----------------------------------------------------MSKKSNK
D.discoideum              NNFNYNKLQFTTQSPNPTPTPTPTPTPTPTPNNNENNNNKNNNNNNFKEEQKKILQEKNK
C.reinhardtii             GPVA-----WS-------AGPAAA----GT--------RS--------AHSRSSTKYTPA
R.americana               ------------------------------------------------------MFKNRK
A.thaliana                KRVF-----WGSSSS-WSLNSHSA----TA--------KSMLDSAHRQYSTHSPSETKSQ
O.sativa                  GL----------ASS-ASSSSSAA----A---------------AAAVAAAAAGREKSSR

R.sphaeroides             KTAGGLVLVVAVMGAASFAAVPFYNWFCRVTGFAGTTAVATEAP---------------A
H.sapiens                 TTLTYVAAVAVGMLGASYAAVPLYRLYCQTTGLGGSAVAGHASDKIENM----------V
D.melanogaster            STLYYITAGGVLIVGLSYAAVPLYSIFCQAYSYGGTTTQGHDAEKVEHM----------K
N.crassa                  STMYYVISVILGTVALSYGSVPMYKMICQTTGWGGQPVRAHGAGGSDSD--VDLAAKLEP
S.cerevisiae              TVAFYFSSVAVLFLGLAYAAVPLYRAICARTGFGGIPITDRR---------KFTDDKLIP
R.prowazekii              NLAFSLLGLMMSMVLLSFASVPIYNLFCKVTGYGGTTIKETVS-VY------------SK
D.discoideum              AIGLYVLIAIIGILGLSYAAVPLYRIFCRATGYGGTTRDADDFDVIRKRNL---------
C.reinhardtii             ELGLYWGAAAVFMVGVSYASVPLYKLFCAATGYGGTVRAGESVEEKLQRRRDAPNAKVEE
R.americana               SIAILIAAVSITMIGFSYGSVPLYRIFCQVTGFGGTTQVADLESDILTL--K------DE
A.thaliana                KMLYYLTAVVFGMVGLTYAAVPLYRTFCQATGYGGTVQRKETVEEKIARHSE------SG
O.sativa                  RTLAYLLGVAAAMVGASYAAVPLYRRFCQATGYGGTVQRRESVEEKISRHAR------DG
                                          ::.:**:*   *   . .*

R.sphaeroides             EVLDRTVKVRFDASREAGMPWEFRPLQREMKLKIGETGLAFYEAYNPTDRTVAGTASYNV
H.sapiens                 PVKDRIIKISFNADVHASLQWNFRPQQTEIYVVPGETALAFYRAKNPTDKPVIGISTYNI
D.melanogaster            KIEDRVLKIRFNADIGSSMRWNFKPQQYEIKVAPGETALAFYTARNPTDKPVIGISTYNV
N.crassa                  VRDAKRMRVTFSASVSDVLPWKFVPQQREVRILPGETALAFYTATNMSDKDIIGVATYSV
S.cerevisiae              VDTEKRIRISFTSEVSQILPWKFVPQQREVYVLPGETALAFYKAKNYSDKDIIGMATYSI
R.prowazekii              VKGTKAIIIEFDANVDPNLPWHFIPRQKRVQIVPGQNTLVFYEAENLSNKDIIGTSIYNV
D.discoideum              DRTVYPIKVTFSASTANKIPWTFKPTQSTIECLPGEPVLCFYRATNNTDTPIIGVATYNI
C.reinhardtii             AASKRELRVWFNADVADDMPWDFRPTQEYVRVRPGQSTLVFFTAHNKSDKPVTGYSLYNV
R.americana               QQENRIITVRFNGDVSDTMPWKFHPIQQEIKVMVGETALAFYSAENPTDSSIIGISTYNV
A.thaliana                TVTEREIVVQFNADVADGMQWKFTPTQREVRVKPGESALAFYTAENKSSAPITGVSTYNV
O.sativa                  TTTSREIIVQFNADVADGMPWKFIPTQREVKVKPGESALAFYTAENRSSAPITGVSTYNV
                                : : * ..    : * * * *  :    *:  * *: * * :.  : * : *.:
```

```
R.sphaeroides       TPDAAGGYFAKIACFCFTEQVLAPGERVEMPVTFYVDPAIIDDPDGRYVRQITLSYTFHE
H.sapiens           VPFEAGQYFNKIQCFCFEEQRLNPQEEVDMPVFFYIDPEFAEDPRMIKVDLITLSYTFFE
D.melanogaster      IPFEAGAYFNKIQCFCFEEQQLNPHEEVDMPVFFYIDPEITADPALETCDTITLSYTFFE
N.crassa            TPGQVAPYFSKIQCFCFEEQRLNAGETVDMPVFFYLDPDYLNDLNMKGIETVTLSYTFFK
S.cerevisiae        APGEAAQYFNKIQCFCFEEQKLAAGEEIDMPVFFFIDPDFASDPAMRNIDDIILHYTFFR
R.prowazekii        TPNKAGKYFVKIHCFCFEEQLLKAREKVLMPVTFYIDNDFERDPEMENIKVITLSYSFFK
D.discoideum        TPMKAGTYFTKIQCFCFDEQRINAHETIDMPVLFVIEPELLDDKNMKGVSDITLSYTFFK
C.reinhardtii       TPDKAAFYFNKIQCFCFEEQRLRPGEQLDMPVFFYVDPEFATDWNCRNINDITLSYVFNK
R.americana         NPQQAGIYFNKIQCFCFEEQRLKPHETIDMPVFFFIDPAILDDPKMSDIDSITLSYTFFN
A.thaliana          TPMKAGVYFNKIQCFCFEEQRLLPGEQIDMPVFFYIDPEFETDPRMDGINNLILSYTFFK
O.sativa            APMKAAIYFNKIQCFCFEEQTLLPGEQIDMPVFFYIDPEFETDPKMEGVNNIVLSYTFFK
                     *  .. ** ** **** ** :   * : *** * ::     *        : * * * .

R.sphaeroides       TALTEEQAALAAESATDVN----------------------------------
H.sapiens           AKEGHKLPVPGYN----------------------------------------
D.melanogaster      AKEGLKLNFPSYA-------KPHAASA--------------------------
N.crassa            AKYDDNGVLKGVPGAP-------------------------------------
S.cerevisiae        AHYGDGTAVSDSKKEPEMNADEKAASLANAAILSPE----VIDTRKDNSN---
R.prowazekii        IREL-------------------------------------------------
D.discoideum        SNDQGEYEEI-------------------------------------------
C.reinhardtii       VEGEEDEEDDGRPSVVKLHSGPHPAAAAAAAVGMPMPVPVAVAAAAGTAPRAA
R.americana         VEDL-------------------------------------------------
A.thaliana          VSEENTTETVNNNNSVPVQETN-------------------------------
O.sativa            VNDS-------------------------------------------------
```

SUPPLEMENTARY FIGURE 4 │ Alignment of COX11 homolog sequences from different species. Sequences were retrieved from GenBank database and aligned. The vertical lines (I) indicate identical residues between the two sequences, while the colons (:) and periods (.) represent conservative substitutions with strongly and weakly similar properties, respectively. Cysteines are labelled yellow. The putative copper-binding motifs are boxed. Predicted transmembrane domains are in grey. Species were chosen to represent all kingdoms. *Rhodobacter sphaeroides* (*Proteobacteria*, purple bacteria), *Homo sapiens* (human), *Drosophila melanogaster* (fruit fly), *Neurospora crassa* (*Ascomycota*, red bread mold), *Saccharomyces cerevisiae* (baker's yeast), *Rickettsia prowazekii* (gram-negative alpha-*Proteobacteria*), *Dictyostelium discoideum* (*Mycetozoa*, slime mold), *Chlamydomonas reinhardtii* (single-cell green alga), *Reclinomonas americana* (protist in the class *Jacobea*), *Arabidopsis thaliana* (dicot plant), *Oryza sativa* (rice, monocot plant).
