# Supplementary material for: The Arabidopsis COX11 Homolog is Essential for Cytochrome c Oxidase Activity
Source: Front Plant Sci. 2015 Dec 18;6:1091. doi: 10.3389/fpls.2015.01091 (PMC4683207; doi:10.3389/fpls.2015.01091)
Supplement: Supplementary file 10 [file Image5.PDF]

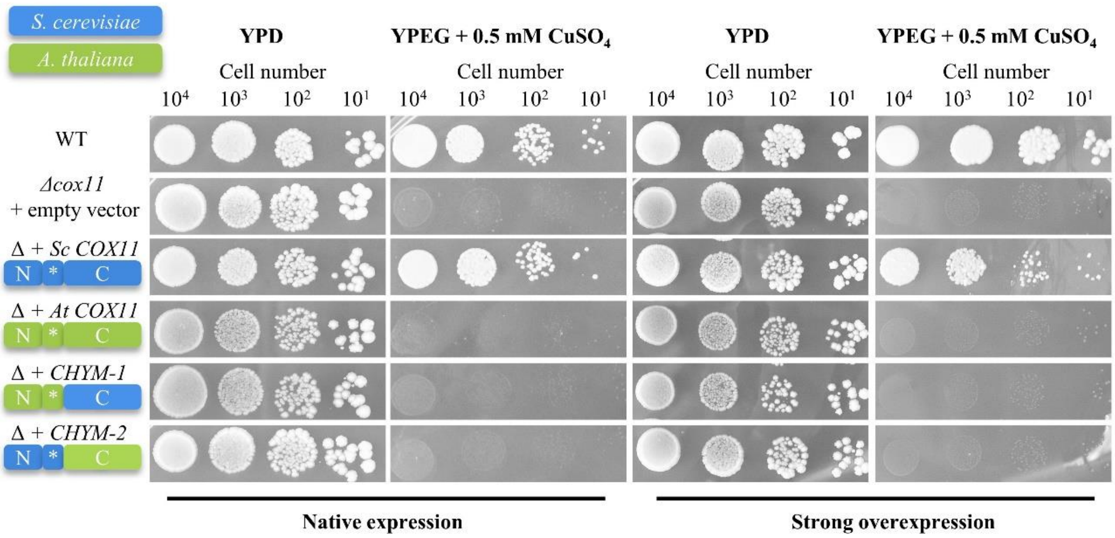

SUPPLEMENTARY FIGURE 5 │Respiratory competence test of yeast *Δcox11* stains expressing full-length yeast and *Arabidopsis COX11* and two chimeric proteins (CHYM-1 and -2). Proteins were expressed under either native conditions (yeast *COX11* promoter, single copy vector) or highly overexpressing conditions (strong *ADH* promoter from multicopy vector). Schematic diagrams of tested proteins depict their structure (N – N-terminal region, C – C-terminal region, * – transmembrane domain) and the origin of the respective *COX11* homolog (blue *S. cerevisiae*; green *A. thaliana*). Serial dilutions of yeast strains were spotted on fermentable glucose media (YPD) or respiratory media (YPEG, ethanol + glycerol media supplemented with 0.5 mM $CuSO_4$).
