# Supplementary material for: The Arabidopsis COX11 Homolog is Essential for Cytochrome c Oxidase Activity
Source: Front Plant Sci. 2015 Dec 18;6:1091. doi: 10.3389/fpls.2015.01091 (PMC4683207; doi:10.3389/fpls.2015.01091)
Supplement: Supplementary file 11 [file Image6.PDF]

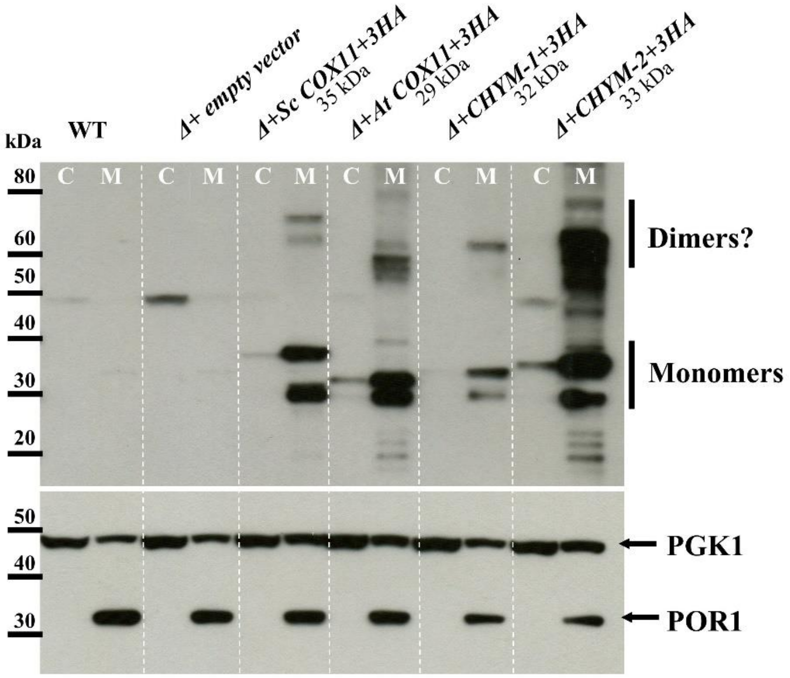

SUPPLEMENTARY FIGURE 6 │ Subcellular localisation of 3HA tagged COX11 proteins. Yeast strains were sub-fractionated into two crude fractions: C (cytoplasm) and M (mitochondria) and analysed by Western blot. 3HA tagged COX11 proteins (theoretical molecular weight after cleavage of the predicted targeting signal ranging from 29 to 35 kDa) were detected with an HA- antibody. The purity of the fractions was evaluated with antibodies against cytoplasmic and mitochondrial marker proteins (PGK1; phosphoglycerate kinase 1, 44.7 kDa and POR1; Porin 1, 30.4 kDa, respectively). All proteins were detected on one membrane, which was stripped only after first detection.
