# Supplementary material for: The Arabidopsis COX11 Homolog is Essential for Cytochrome c Oxidase Activity
Source: Front Plant Sci. 2015 Dec 18;6:1091. doi: 10.3389/fpls.2015.01091 (PMC4683207; doi:10.3389/fpls.2015.01091)
Supplement: Supplementary file 12 [file Image7.PDF]

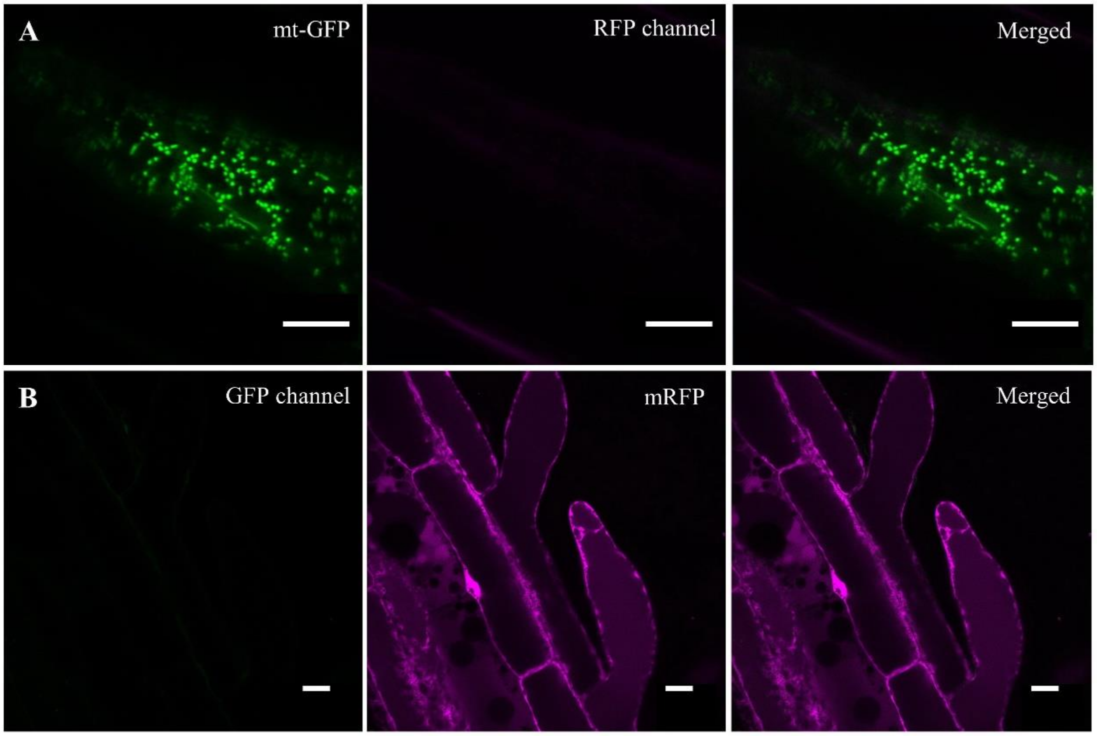

SUPPLEMENTARY FIGURE 7 │ Bleed-through was not observed for GFP and mRFP. CLSM images of root cells from *Arabidopsis* expressing GFP targeted to mitochondria (*mt-GFP*) **(A)** or *mRFP* **(B).** The fluorescence of mt-GFP and mRFP was falsely coloured green and magenta, respectively. Scale bars correspond to 10 µm.
