# Supplementary material for: The Arabidopsis COX11 Homolog is Essential for Cytochrome c Oxidase Activity
Source: Front Plant Sci. 2015 Dec 18;6:1091. doi: 10.3389/fpls.2015.01091 (PMC4683207; doi:10.3389/fpls.2015.01091)
Supplement: Supplementary file 13 [file Image8.PDF]

A

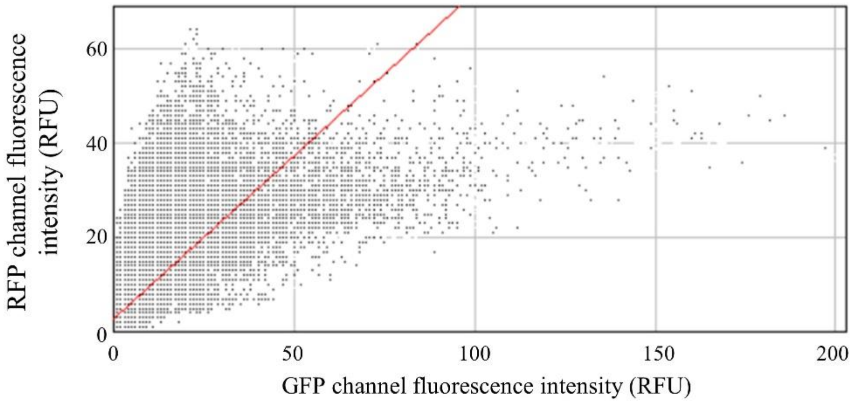

B

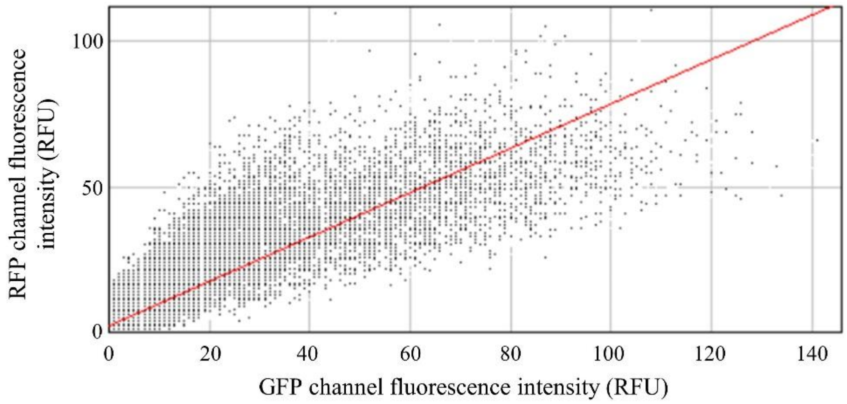

SUPPLEMENTARY FIGURE 8 | Scatter plot analysis of COX11-mRFP colocalization with mt-GFP. Each dot on the graph represents one pixel from the analyzed image and the dot's position reflects the pixel's fluorescence intensity for both channels (RFU – relative fluorescence units). The fitted red line represents the correlation between the fluorescence intensities of the two channels. **(A)** Scatter plot of the image depicted in **Figure 2A** (root cell expressing *mt-GFP* and *mRFP*). In the plot, pixels cluster towards the axes, indicating absence of colocalization. **(B)** Scatter plot of the image depicted in **Figure 2B** (root cell expressing *mt-GFP* and *COX11-mRFP*). In the plot, pixels cluster in the middle, indicating similar fluorescence intensity in both channels and colocalization.
