# Supplementary material for: The Arabidopsis COX11 Homolog is Essential for Cytochrome c Oxidase Activity
Source: Front Plant Sci. 2015 Dec 18;6:1091. doi: 10.3389/fpls.2015.01091 (PMC4683207; doi:10.3389/fpls.2015.01091)
Supplement: Supplementary file 14 [file Image9.PDF]

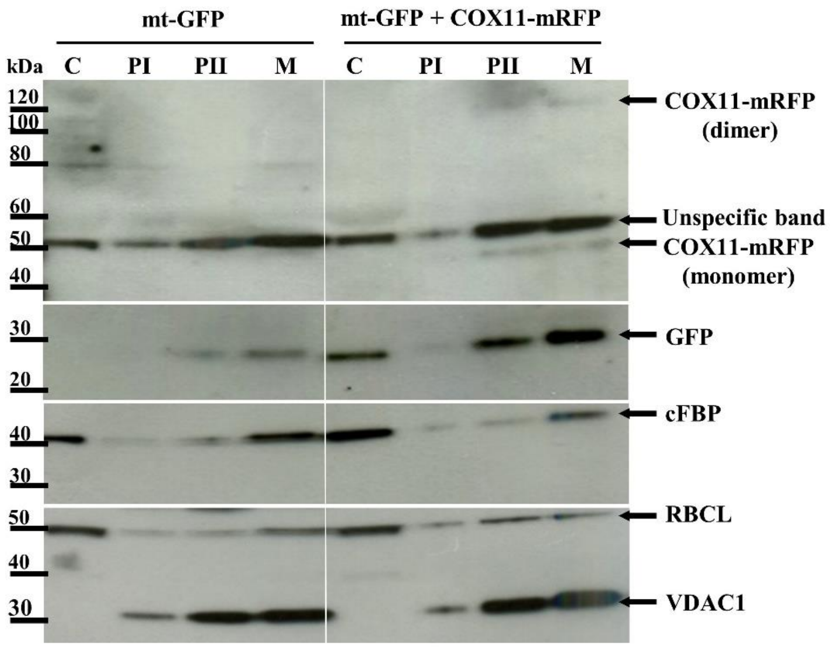

SUPPLEMENTARY FIGURE 9│The fusion protein COX11-mRFP is not cleaved and is targeted to mitochondria. Etiolated seedlings, expressing *mt-GFP* only (left panel) or *mt-GFP* and *COX11-mRFP* (right panel) were sub-fractionated into four crude fractions: C (cytoplasm) PI (pellet I; etioplasts, nuclei), PII (pellet II; mostly etioplasts and mitochondria) and M (mitochondria) and analysed by Western blot. COX11-mRFP (theoretical molecular weight of 50 kDa after cleavage of the predicted targeting signal) was detected with an mRFP-specific antibody. The GFP antibody was used to detect the mt-GFP (~30 kDa). The purity of the fractions was evaluated with antibodies against cytoplasmic, etioplast and mitochondrial marker proteins (cFBP; cytoplasmic fructose bisphosphatase, 37 kDa; RbCL; RuBisCO large subunit, 53 kDa and VDAC1; voltage-dependent anion channel 1, 29 kDa, respectively). All proteins were detected on one membrane, which was stripped after each detection.
