# Supplementary material for: The Arabidopsis COX11 Homolog is Essential for Cytochrome c Oxidase Activity
Source: Front Plant Sci. 2015 Dec 18;6:1091. doi: 10.3389/fpls.2015.01091 (PMC4683207; doi:10.3389/fpls.2015.01091)
Supplement: Supplementary file 15 [file Image10.pdf]

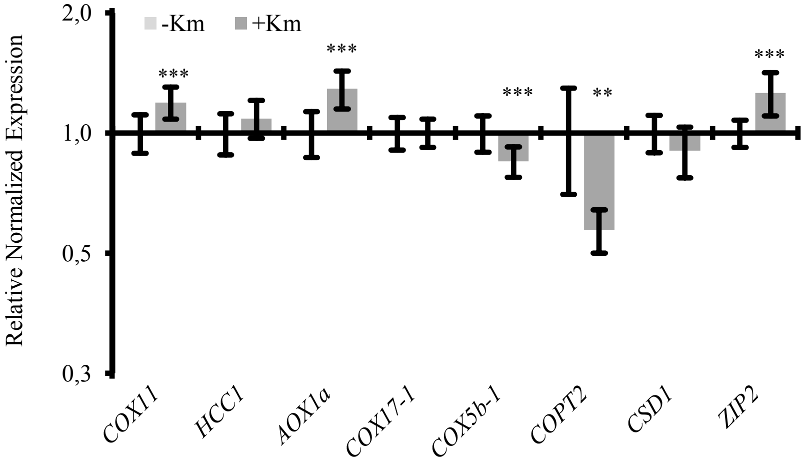

SUPPLEMENTARY FIGURE 10 | Effect of kanamycin on mRNA levels of genes of interest. qPCR analysis of expression levels of various genes of interests in kanamycin (Km)-resistant *Arabidopsis* seedlings. The seedlings were grown for 14 days in the absence or presence of Km. They represent a line homozygous for the *35S:GUS* construct. Mean values of mRNA levels in Km-treated seedlings were normalized to control seedlings and plotted on a logarithmic scale (base two). Values and statistical significance (compared with the control, $^{**}P < 0.01$, $^{***}P < 0.001$) were calculated with the CFX manager software. Error bars represent ± SD. Individual values are listed in the **Supplementary Table 5**.
