# Supplementary material for: The Arabidopsis COX11 Homolog is Essential for Cytochrome c Oxidase Activity
Source: Front Plant Sci. 2015 Dec 18;6:1091. doi: 10.3389/fpls.2015.01091 (PMC4683207; doi:10.3389/fpls.2015.01091)
Supplement: Supplementary file 16 [file Image11.pdf]

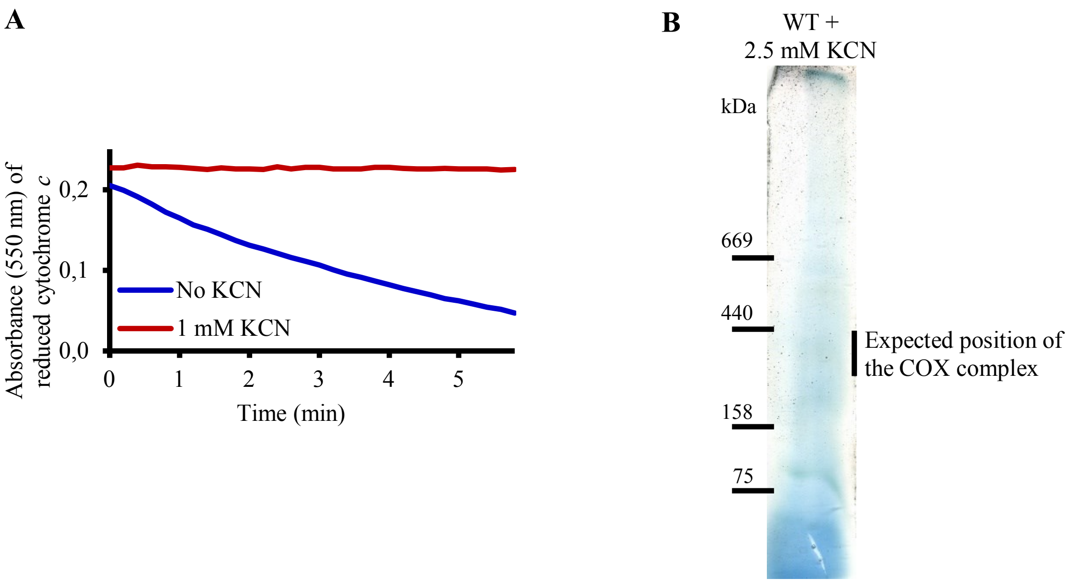

SUPPLEMENTARY FIGURE 11 | Specific inhibition of COX complex activity by KCN. (A) Exemplary COX activity measurement with 10 µg of WT mitochondrial protein in the presence (red line) or absence (blue line) of 1 mM KCN. **(B)** Mitochondrial complexes from WT plants were separated by gradient BN-PAGE and subsequently stained in gel for COX complex activity in the presence of 2.5 mM KCN, as a control for the specificity of the activity staining.
