# Supplementary material for: The Arabidopsis COX11 Homolog is Essential for Cytochrome c Oxidase Activity
Source: Front Plant Sci. 2015 Dec 18;6:1091. doi: 10.3389/fpls.2015.01091 (PMC4683207; doi:10.3389/fpls.2015.01091)
Supplement: Supplementary file 17 [file Image12.PDF]

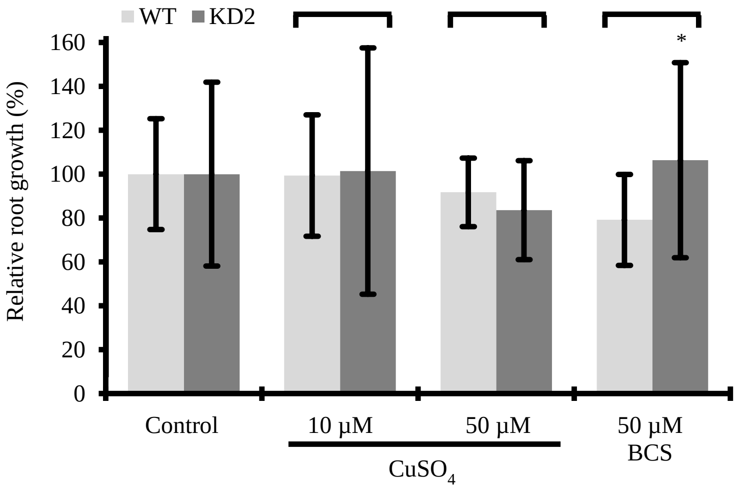

SUPPLEMENTARY FIGURE 12 │ Relative root growth of 12-day-old WT and KD2 seedlings cultured on plates with copper excess or deficiency. Root length is expressed relative to WT or mutant seedlings on control plates. Roots were measured in three independent experiments. Error bars represent ± SD for n = ~30 (WT) and 10-15 (KD2). Asterisks indicate statistical significance calculated with the Student's t test (mutant line compared with WT, $^{*}P < 0.05$). Individual values are listed in the **Supplementary Table 5**. BCS - bathocuproinedisulfonic acid.
