# Supplementary material for: The Arabidopsis COX11 Homolog is Essential for Cytochrome c Oxidase Activity
Source: Front Plant Sci. 2015 Dec 18;6:1091. doi: 10.3389/fpls.2015.01091 (PMC4683207; doi:10.3389/fpls.2015.01091)
Supplement: Supplementary file 18 [file Image13.pdf]

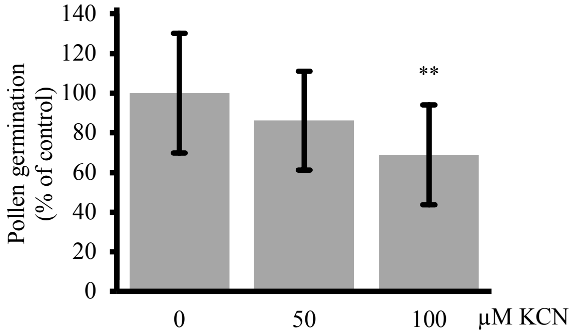

SUPPLEMENTARY FIGURE 13 | Infuence of KCN on pollen germination. Germination rates of WT pollen treated with KCN, normalized to the untreated control (= 100%). Error bars represent ± SD of means from five experiments. For each experiment, germination of 100 – 300 pollen grains was evaluated in duplicates or triplicates. Asterisks indicate statistical significance calculated with the Student's *t* test (**$P < 0.01$). Exact values are given in **Supplementary Table 5.**

.
